# Playback of Alarm and Appetitive Calls Differentially Impacts Vocal, Heart-Rate, and Motor Response in Rats

**DOI:** 10.1016/j.isci.2020.101577

**Published:** 2020-09-19

**Authors:** Krzysztof H. Olszyński, Rafał Polowy, Monika Małż, Paweł M. Boguszewski, Robert K. Filipkowski

**Affiliations:** 1 Behavior and Metabolism Research Laboratory, Mossakowski Medical Research Centre, Polish Academy of Sciences, 5 Pawinskiego St, 02-106 Warsaw, Poland; 2 Laboratory of Animal Models, Nencki Institute of Experimental Biology, Polish Academy of Sciences, 3 Pasteur St, 02-093 Warsaw, Poland

**Keywords:** Biological Sciences, Animals, Ethology

## Abstract

Our rudimentary knowledge about rat intraspecific vocal system of information exchange is limited by experimental models of communication. Rats emit 50-kHz ultrasonic vocalizations in appetitive states and 22-kHz ones in aversive states. Both affective states influence heart rate. We propose a behavioral model employing exposure to pre-recorded playbacks in home-cage-like conditions. Fifty-kHz playbacks elicited the most vocalizations (>60 calls per minute, mostly of 50-kHz type), increased heart rate, and locomotor activity. In contrast, 22-kHz playback led to abrupt decrease in heart rate and locomotor activity. Observed effects were more pronounced in singly housed rats compared with the paired housed group; they were stronger when evoked by natural playback than by corresponding artificial tones. Finally, we also observed correlations between the number of vocalizations, heart rate levels, and locomotor activity. The correlations were especially strong in response to 50-kHz playback.

## Highlights

• Rats vocalized abundantly when exposed to recorded ultrasonic playback
• Playback of appetitive calls caused an increase in heart rate
• Playback of alarm/aversive calls caused a decrease in heart rate
• Singly housed rats exhibited more vocalizations and higher heart rate

## Graphical Abstract

**Figure:**
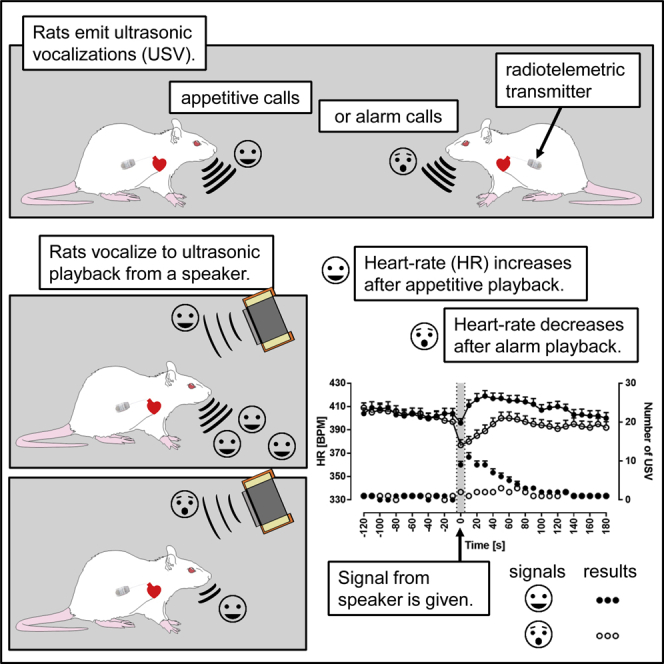

## Introduction

Ultrasonic vocalizations (USV) of adult rats are divided into two main categories: 22-kHz and 50-kHz USV. The former usually accompany aversive situations (fear, anxiety, social aggression, predator scent/presence, unfamiliar humans, air-puff). The latter are emitted in appetitive contexts such as rough and tumble play, social exploration, reward anticipation and acquisition, successful copulation, and tickling (Wohr and Schwarting, 2013; Simola and Granon, 2019; Burgdorf et al., 2020).

Suitable behavioral models to investigate vocal communication in rats are lacking, with a significant problem in identifying the USV emitter when more than one animal is present. Attempts to solve the problem require elaborate technical solutions that fail to unambiguously assign all USV (Neunuebel et al., 2015) or require invasive devocalization that changes social dynamics between rats (White and Barfield, 1989; Kisko et al., 2015a, 2015b). However, playback studies, i.e. with the presentation of recorded or prepared calls, can offer some simplification by replacing one of the animals with a speaker. Nonetheless, they proved not to be effective to evoke USV emissions (White and Barfield, 1989; White et al., 1993; Wohr and Schwarting, 2007, 2009; Sadananda et al., 2008; Berg et al., 2018). To this end, we wanted to first verify whether rats, under low stress experimental conditions, would vocalize to pre-recorded USV played from a speaker.

Previous playback studies have shown that presenting 50-kHz USV evoked various behaviors such as approach (Wohr and Schwarting, 2007), positive responses to ambiguous cues (Saito et al., 2016), reduction in fights (Kisko et al., 2015a), restoration of sexual activity in devocalized rats (White and Barfield, 1989), and awakening from haloperidol-induced catalepsy (Tonelli et al., 2018). Whereas, exposing rats to 22-kHz playback inhibited behavior (Wohr and Schwarting, 2007), decreased locomotor activity during replay and immediately afterward (Sales, 1991; Brudzynski and Chiu, 1995), and caused negative responses to ambiguous cues (Saito et al., 2016), and anxiety-like behaviors (Demaestri et al., 2019). Presentation of 50-kHz versus 22-kHz playback also resulted in activation of c-Fos in different brain areas (Beckett et al., 1997; Sadananda et al., 2008; Ouda et al., 2016; Demaestri et al., 2019).

Finally, there is a social effect of USV emission. Production of 22-kHz USV during and after exposure to a predator increased in the presence of familiar conspecifics, which may serve as alarm cries (Blanchard et al., 1991). Female vocalizations have also been found to affect the sociosexual behavior of male rats (White and Barfield, 1989). Since USV are means of communication, perhaps rodents housed in various social conditions, e.g. single versus paired, would react differently to USV presented. Recent findings show that rats respond to 50-kHz calls differently according to their period of isolation (Seffer et al., 2015).

Heart rate (HR) is a physiological variable that changes substantially in aversive and appetitive situations. Resting HR state is mainly controlled by the autonomic nervous system through the parasympathetic inhibition *via* the vagus nerve. Notably, lowering the vagal tone in the heart and increasing the sympathetic tone correlates with chronic stress, emotional trauma, and anxiety in humans (Carnevali and Sgoifo, 2014). Moreover, regulation of HR, respiratory rhythm, vocal emissions, and other behaviors are controlled by the same brain areas, e.g. the nucleus of the solitary tract (afferent path), the nucleus ambiguus (efferent path), and also a common signaling pathway—multiple neural pathways of the vagus nerve—as described by the polyvagal theory (Porges, 1995, 2007, 2009).

In this study, we investigated (1) whether hearing recorded USV from a speaker can lead to vocal, motor, and HR response in rats; (2) the direction of HR changes in response to 50-kHz versus 22-kHz playback presented as unrelated to behavioral situation; (3) whether rats can distinguish between artificially-generated tones and natural USV, i.e. by measuring distance traveled, vocal, and cardiovascular responses; (4) role of social context in response to ultrasonic playback, i.e. paired versus singly housed rats; and finally (v) whether these variables correlate.

## Results

### Except for the Periods of Ultrasonic Playback, Rats' Behavior Remained Relatively Constant

**Figure 1:**
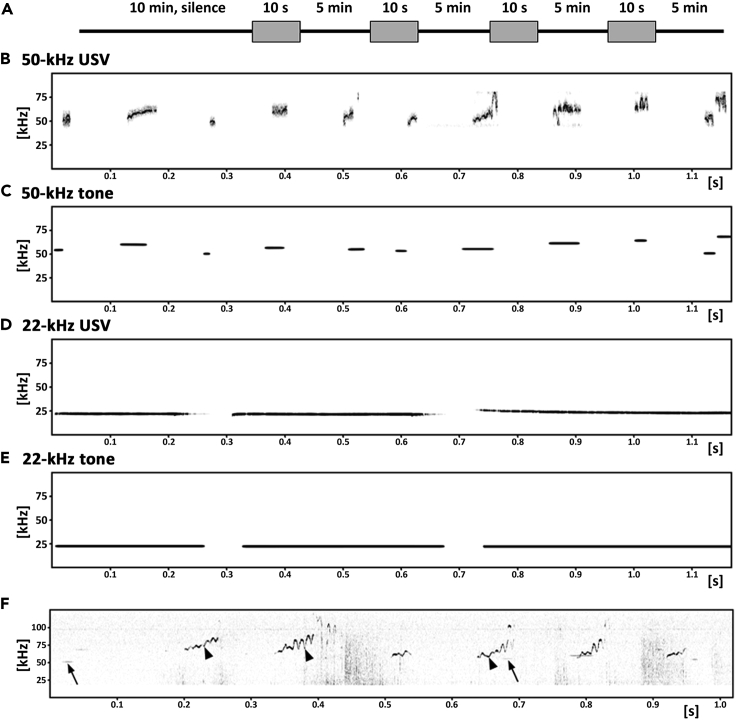
Scheme of the Experiment (A–E) Scheme of experiment (A) with motives of ultrasonic signals (B–E) played during playback sessions (gray rectangles). After 10-min silence, four different signals were presented to each rat in counterbalanced order. Each set of 10-s signals was followed by 5 min of silence; (B and D) natural USV, i.e. recorded from animals; (C and E) artificial software-generated signals imitating natural ones, i.e. with the same duration, inter-signal interval, mean peak frequency, and amplitude. (F) Example of a spectrogram with rat ultrasonic vocalizations (USV, black arrowheads) recorded during 50-kHz tone playback (black arrows).

**Figure 2:**
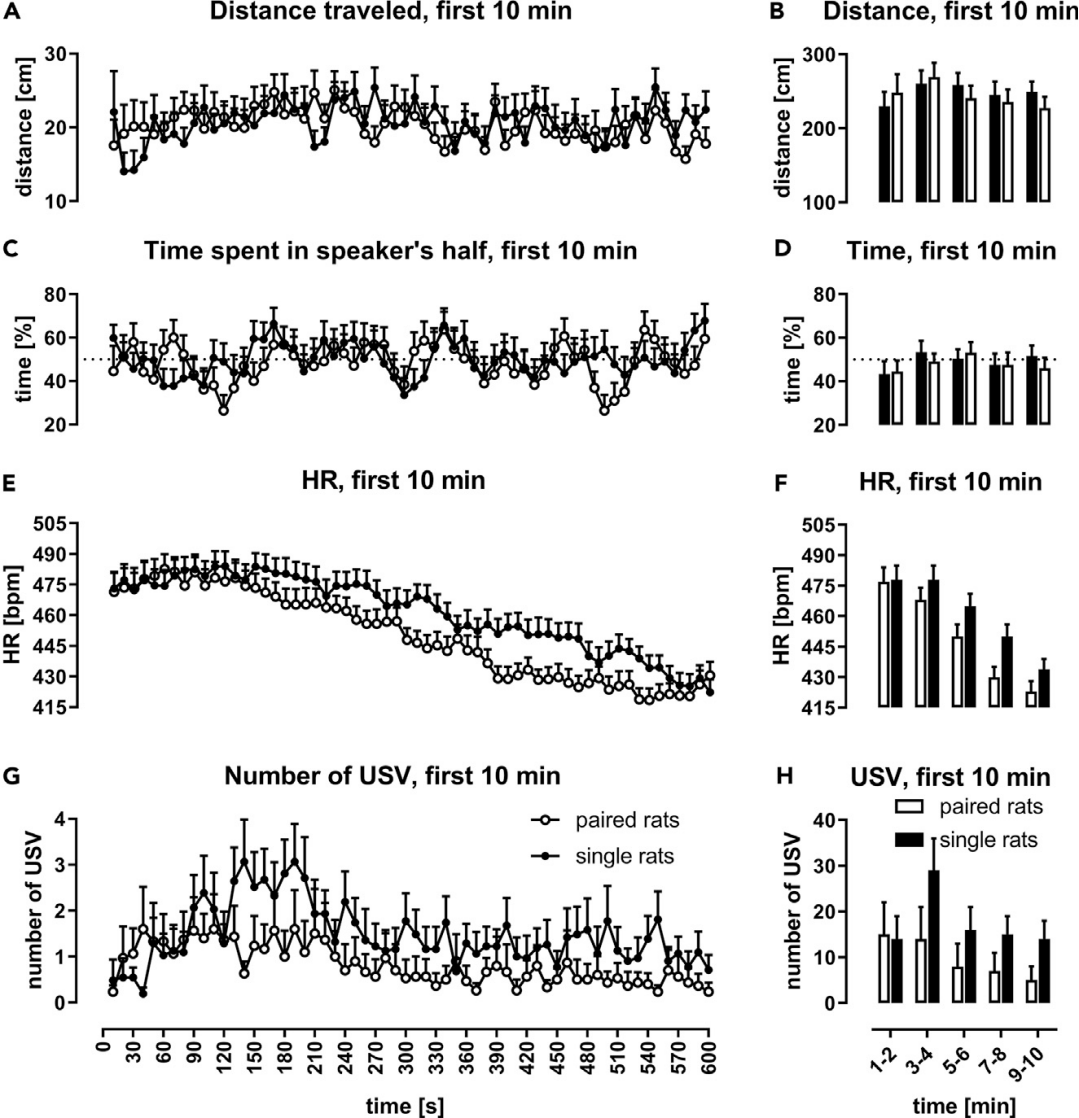
Assessment of Locomotor Activity, Heart Rate (HR), and the Number of USV Emitted of All Animals during the First 10 min of Silence in the Experimental Cage Shown are (A and B) distance traveled (cm); (C and D) time (%) spent in a speaker's half of the cage with horizontal dotted line marking 50% chance level; results above it show more time in the speaker's half; (E and F) heart rate (bpm; beats per minute); (G and H) number of USV. HR declined progressively in both groups; however, this decrease was more pronounced in paired rats; single rats vocalized more than paired rats. Mean values with SEM are presented for 10-s intervals in the line graphs (A, C, E, and G) and 2-min intervals in bar graphs (B, D, F, and H). Values for paired rats (n = 29/30) are presented as blank dots/bars and for single rats (n = 31) as solid dots/bars; for p values see Tables S1–S6.

**Figure 3:**
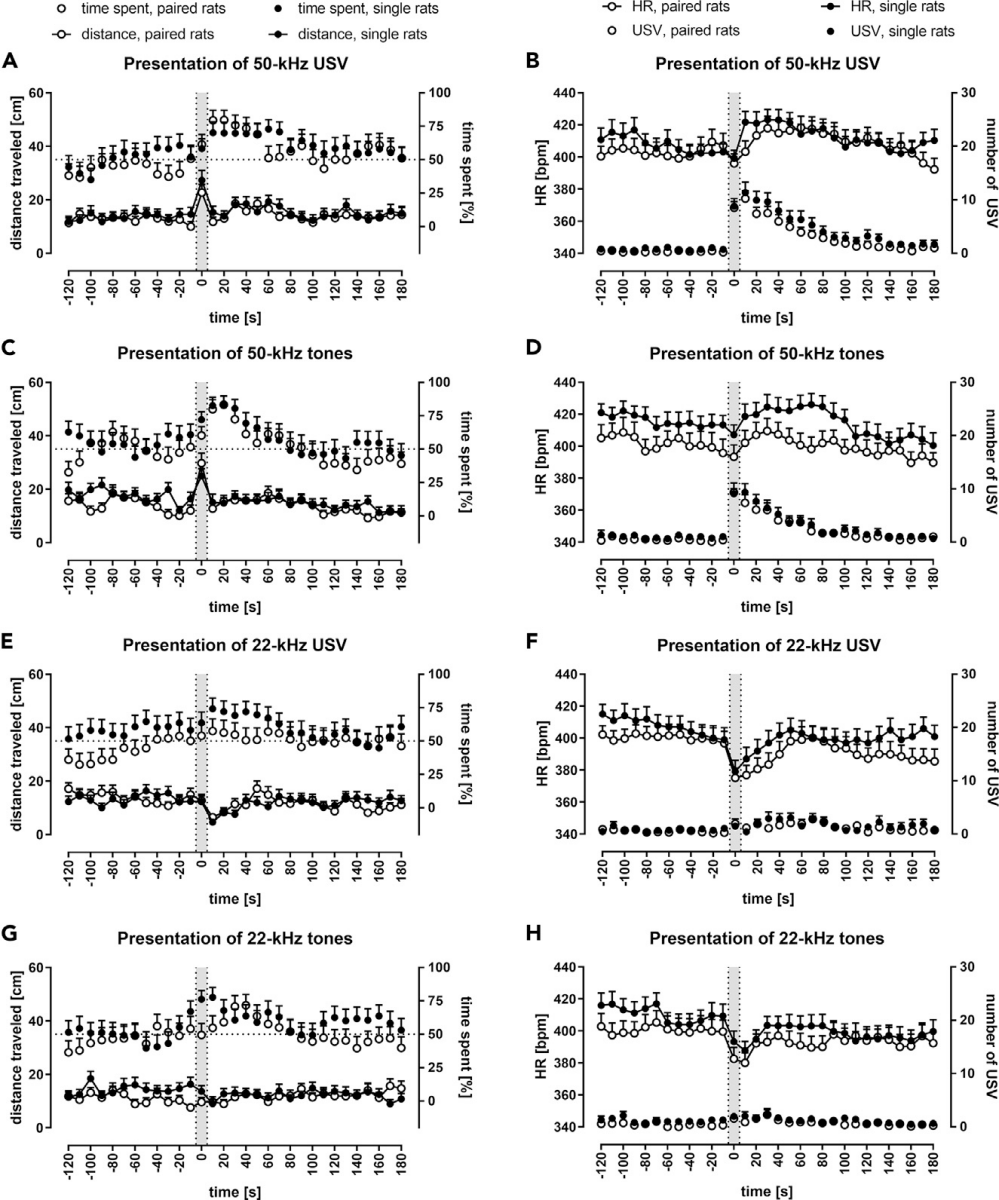
Response to Appetitive versus Aversive Playback Impact of ultrasonic playback on distance traveled, time spent in the speaker's half of the cage, heart rate (HR), and USV emission. Gray sections correspond to the 10-s-long ultrasonic presentation. Graphs depict responses after exposure to: 50-kHz USV. (A, B), 50-kHz tones (C, D), 22-kHz USV (E, F), and 22-kHz tones (G, H). In the left column (A, C, E, G), distance traveled is presented as connected dots (cm, left Y axis) and percentage of time spent in the speaker's cage half as not connected dots (%, right Y axis). In the right column (B, D, F, H), HR is presented as connected dots (bpm; beats per minute, left Y axis); the number of USV is presented as not connected dots (right Y axis). Each point is a mean for a 10-s-long time-interval with SEM. The dotted horizontal line marks a 50% chance value for time in a side of the cage. HR tended to be higher in single (lines with solid dots) than in paired rats (blank dots); 50-kHz playback increased locomotor activity in both paired and single rats. Each ultrasonic playback caused HR changes in all groups; paired rats, n = 29/30; single, n = 31.

Locomotor activity, measured as distance traveled, was the same during the 10-min silence period (Figure 1A) at an average speed of 2 cm/s (Figures 2A and 2B; Tables S1A and S3), which declined during the playback session to 1 cm/s (Figures 3A, 3C, 3E, and 3G). See also control time-intervals, from −120 s to −100 s and −30 s to −10 s (Tables S7A and S8A). We did not observe a strong preference for either side of the cage during the initial 10 min (Figures 2C and 2D), although the values fluctuated (Tables S1B and S4). In general, the rats did not prefer a cage-side during the sound presentation sessions; please note the values around 50% (dotted line) before each ultrasonic emission (Figures 3A, 3C, 3E, and 3G).

### Single Rats Behaved Differently Than Paired Ones

The single rats usually, i.e. during the majority of time-intervals, traveled longer distances than the paired ones during the first 10 min of silence (Figures 2A and 2B; Tables S2A and S3) as well as during ultrasound presentation session (Figures 3A, 3C, 3E, and 3G; Tables S9 and S13A), the effect was not significant in most cases, i.e. for particular time-intervals; see, however, 4 cases for 50-kHz tone playback, Table S9. The single rats initially spent the same amount of time in both sides of the cage as did the paired rats during the introductory silence (Figures 2C and 2D; Table S4) but tended to spend more time in the speaker's half of the experimental cage during the playback session compared with the paired rats (Figures 3A, 3C, 3E, and 3G; Tables S10 and S13B). However, this preference for the speaker's side of the cage in the single rat group was not always statistically significant (5 cases in Table S13B).

### HR Levels Declined during the Whole Experimental Session

When average levels of HR from the first 5 min of the 10-min silence period were compared with those from the last 5 min of the playback session (Figure 1A), there was a significant decline in the paired (467.7 ± 5.9 versus 377.5 ± 6.3, p < 0.001), single (476.3 ± 6.2 versus 384.4 ± 6.1, p < 0.001), and all rats (472.1 ± 4.3 versus 381.0 ± 4.4, p < 0.001; all Wilcoxon). The decline began at around 180 s and continued for rest of the 10 min in all groups (Figures 2E and 2F, and S1C, and Table S5, p < 0.001, Friedman). When HR levels were compared with the very first time-interval, i.e. 10 s, the significant decline started from 280 to 350 s onward (Table S5).

### Single Rats Displayed Higher HR Levels throughout the Experiment

The HR of single and paired rats began to differ during the second half of the 10-min silence period (Figures 2E and 2F) such that the HR of single rats was significantly higher in 14 of the 10 s time-intervals measured (Tables S2A and S5). Moreover, HR of the single rats remained higher than the paired rats throughout the playback session (Figures 3B, 3D, 3F, and 3H; Tables S11 and S14C), with significant differences at some time-intervals (4 cases for Table S11; 5 cases for Table S14C), notably at 10 s after the playback of 50-kHz USV as well as both 50-kHz USV and tones (Figures 3B and 3D; Table S11, p < 0.05; Table S14C, p < 0.01, Mann-Whitney).

### Single Rats Emitted USV More Often Than Paired Rats

It was observed during the first 10-min silence period (Figures 2G and 2H; Table S2D), especially from around 120 s onward (Table S6). Moreover, when non-vocalizers and outliers were excluded from the analysis of the first 10 min period, the single rats were found to emit more USV at all time-intervals (Figures S1C and S1D; Table S6) and during the whole silence period (Table 1). Most of these USV were 50-kHz USV, the main type of vocalizations emitted throughout the whole experiment, i.e. in >90% of cases (Table 1). The single rats also emitted more USV than the paired rats during the playback session itself (Figures 3B, 3D, 3F, and 3H; Tables S12 and S14D), with several significant differences (Table S14D).

**Table 1:** Average Number of Emitted USV of Different Types and Selected Characteristics of 50-kHz USV Emitted during the Whole Experiment, First 10 min of Silence, and during Playback Sessions, i.e. during the 10-s-Long Playback and 110 s Afterward

Groups	Number of USV				Parameters of 50-kHz USV	
	Total USV	50-kHz USV	Short 22-kHz USV	Long 22-kHz USV	Duration [ms]	MPF [kHz]
**USV emitted during the whole experiment**						

Paired	232.7 ± 47.2	224.9 ± 47.2	5.5 ± 1.6	2.3 ± 1.3	30.3 ± 1.1	58.8 ± 0.6
Single	340.4 ± 67.8	320.5 ± 68.6	5.1 ± 1.5	4.1 ± 4.1	33.0 ± 1.6	60.2 ± 0.8
p value	0.4531	0.7022	0.7905	0.1724	0.2428	0.1984

**USV emitted during the first 10 min of silence**						

Paired	48.9 ± 25.7	46.1 ± 25.6	1.8 ± 0.5	1.0 ± 1.0	19.6 ± 1.5	57.2 ± 1.2
Single	87.6 ± 21.2	81.3 ± 21.3	2.3 ± 0.8	4.1 ± 4.1	20.6 ± 1.6	57.1 ± 1.6
p value	0.0550	0.1687	0.8451	1.0000	0.6241	0.6745

**USV emitted during the first 10 min of silence with non-vocalizers and outliers eliminated**						

Paired	10.6 ± 1.7	8.8 ± 1.8	1.9 ± 0.7	0.0 ± 0.0	19.1 ± 1.8	56.4 ± 1.3
Single	75.4 ± 16.4	67.7 ± 16.6	2.7 ± 1.0	5.1 ± 5.2	19.8 ± 1.6	56.9 ± 1.8
p value	p < 0.01	p < 0.05	0.3568	0.3594	0.6503	0.6686

**USV emitted to 50-kHz USV playback (0–120 s time-intervals)**						

Paired	60.2 ± 9.5	59.8 ± 9.4	0.4 ± 0.2	0.0 ± 0.0	33.1 ± 1.3	59.2 ± 0.5
Single	77.9 ± 14.0	77.7 ± 13.9	0.1 ± 0.1	0.0 ± 0.0	36.2 ± 1.9	59.2 ± 0.7
p value	0.6651	0.6651	0.6020	1.0000	0.1965	0.9133

**USV emitted to 50-kHz tone playback (0–120 s time-intervals)**						

Paired	48.7 ± 8.6	48.4 ± 8.6	0.3 ± 0.2	0.0 ± 0.0	32.5 ± 1.6	58.9 ± 0.9
Single	56.5 ± 10.1	56.3 ± 10.2	0.2 ± 0.1	0.0 ± 0.0	35.7 ± 1.4	60.2 ± 0.6
p value	0.6337	0.6389	0.5548	1.0000	0.1574	0.2023

**USV emitted to 22-kHz USV playback (0–120 s time-intervals)**						

Paired	20.4 ± 6.0	19.2 ± 6.1	1.1 ± 0.8	0.1 ± 0.1	26.1 ± 1.6	58.6 ± 0.7
Single	21.2 ± 5.9	21.0 ± 5.9	0.1 ± 0.1	0.0 ± 0.0	29.5 ± 2.1	60.5 ± 0.9
p value	0.5168	0.3258	0.5841	0.5253	0.3376	0.1939

**USV emitted to 22-kHz tone playback (0–120 s time-intervals)**						

Paired	13.3 ± 4.6	12.4 ± 4.6	0.4 ± 0.3	0.4 ± 0.4	26.9 ± 2.6	56.3 ± 1.6
Single	19.0 ± 5.0	18.8 ± 5.0	0.2 ± 0.1	0.0 ± 0.0	25.7 ± 2.3	57.6 ± 1.7
p value	0.6292	0.4021	0.6295	0.1472	0.7345	0.6387

MPF, mean peak frequency.

The number of USV from three categories: 50 kHz USV (MPF >32 kHz), short 22 kHz (MPF of 18–32 kHz, duration <0.3 s), long 22 kHz (MPF of 18–32 kHz, duration >0.3 s); paired, n = 30; single, n = 31; p values of Mann-Whitney test.

### Animals Moved Faster during 50-kHz Ultrasonic Presentations and Slowed Down after 22-kHz Ultrasonic Presentations

Both the single and paired groups traveled significantly longer distances during the presentation of 50-kHz signals (Figures 3A and 3C; Videos S1 and S2), for both USV and tone playbacks, i.e. at 0 s time-interval versus neighboring −10 s and 10 s time-intervals (Tables S7A and S9). In case of 22-kHz USV playback (Figure 3E; Tables S7A and S9), a reduction in distance traveled appeared immediately after signal presentation, i.e. at 10 s time-interval in the paired rats and single rats (p < 0.01, Table S9, Wilcoxon). This deceleration, however, did not occur after the 22-kHz tone presentation in the paired group (Figure 3G; Tables S7A and S9).

Video S1. An Example of a Paired Rat Before (10 s), During (10 s), and After (10 s) Playback of 50-kHz Ultrasonic Vocalizations, Related to Figure 3 Please note the correlation between locomotor activity and ultrasonic emissions.

Video S2. An Example of a Single Rat Before (10 s), During (10 s), and After (10 s) Playback of 50-kHz Ultrasonic Vocalizations, Related to Figure 3 Please note the correlation between locomotor activity and ultrasonic emissions.

**Figure 4:**
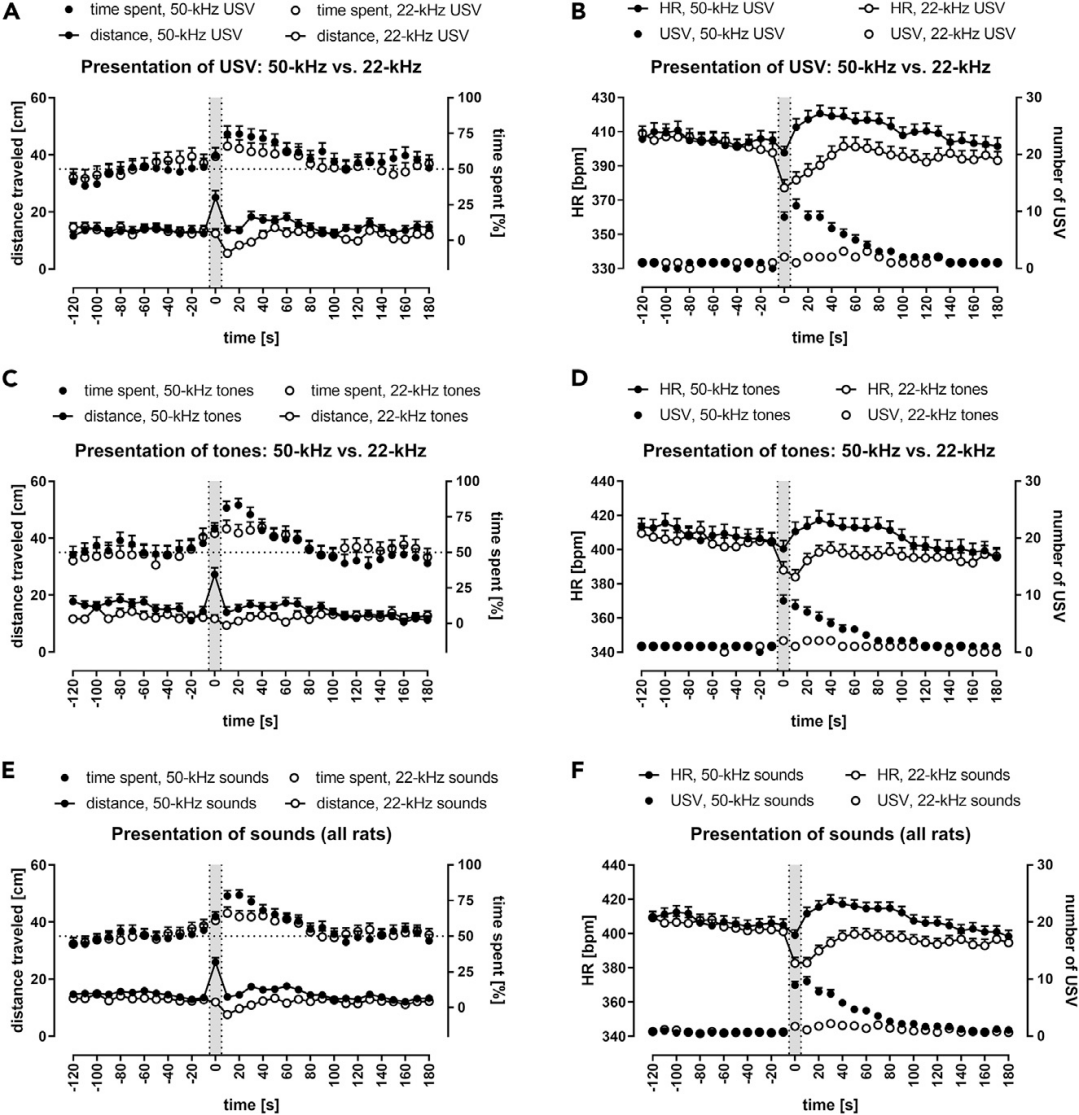
Response to Appetitive versus Aversive Playback (Pooled Data) Comparison of responses to 50-kHz versus 22-kHz playback of USV (A, B) or artificial tones (C, D) in rats pooled together for analysis irrespective of social context, as well as with responses to respective USV and tones pooled together (sounds) for analysis (E, F). Gray sections correspond to the 10-s-long ultrasonic presentations. Responses to 50-kHz sounds are presented as solid dots and to 22-kHz playback as blank dots.(A, C, and E) Distance is presented as connected dots (cm, left Y axis), time spent in the half of the cage proximal to the speaker is presented as not connected dots (%, right Y axis). (B, D, and F) HR is presented as connected dots (bpm; beats per minute, left Y axis); the number of USV is presented as not connected dots (right Y axis). Each data point is a mean for the 10-s-long time-interval with SEM. The dotted horizontal line marks 50% chance value for time spent in the speaker's half of the cage. HR values increased after 50-kHz USV, tones, and sounds and decreased during and after 22-kHz USV, tones, and sounds (B, D, F). The number of USV (B, D, F) and distance traveled (A, C, E) increased during 50-kHz presentations; for p values see Tables S7–S17; n = 60 for A, C; n = 61 for B, D; n = 120 for E; n = 122 for F.

When the distance traveled data from paired and single rats, as well as effects of USV and tones playback (sounds), were pooled together (Figure 4E; Table S8A), we found that rats traveled the most during the presentation of the 50-kHz signal at the 0 s time-interval (significantly more than all other time-intervals, Table S13A). In contrast, the rats moved the least at 10 s, just after the 22-kHz signals playback (Table S13A). Altogether, the distance traveled after 50-kHz playback was significantly higher than that after 22-kHz presentation (Table S15A).

Rats presented with 50-kHz playback spent more time in the speaker's half of the cage than animals exposed to 22-kHz sounds (Figures 4A, 4C, 4E, Figures S2, S2C, S2E, and S2G). The difference was significant for 10–30 s time-intervals for all results pooled together for analysis (Table S15B).

### 50-kHz Sounds Caused HR to Increase; 22-kHz Sounds Caused HR to Decrease

Every presented ultrasonic playback affected HR values in all groups, i.e. single, paired (analyzed separately, Figures 3B, 3D, 3F, and 3H), and pooled groups (analyzed together, Figures 4B and 4D) when presented with 50-kHz USV, 50-kHz tones, 22-kHz USV, and 22-kHz tones (Table S11) as revealed by the analysis of repeated measures for −120 s to 180 s time-intervals (Table S7C, p < 0.001 for all 12 cases, Friedman). Since these differences could be due to the tendency of HR levels to decline over a longer time span, we investigated shorter intervals around the onset of the ultrasonic signal. We found that the HR levels changed significantly, e.g. from −60 s to 60 s time-intervals and, especially, from −10 s to 10 s (at least p < 0.05 for all conditions), compared with the steady HR level before the signal onset, i.e. from −30 s to −10 s or −120 s to −100 s. These HR changes were even more pronounced and significant when the paired and single animals were pooled together for analysis of USV presentations and tone presentations (Table S8C).

The changes in HR around the signal onset, i.e. from −10 s to 10 s, were most striking, especially in rats exposed to 50-kHz versus 22-kHz playback. The former experienced a significant increase in HR values between 0 s time-interval and following time-intervals (Figures 3B and 3D; Table S11), whereas after 22-kHz sounds presentation, the most striking feature was a drop in HR levels between −10 s time-interval and subsequent 0 s and 10 s time-intervals (Figures 3F and 3H; Table S11).

Moreover, when the single and paired rats were analyzed together (Table S11; Figures 4B and 4D), and when comparing both 50 kHz and both 22 kHz groups of results, i.e. USV and artificial tones, with both single and paired rats pooled together (Table S14C; Figure 4F), the tendencies of HR levels to increase or decrease intensified.

**Figure 5:**
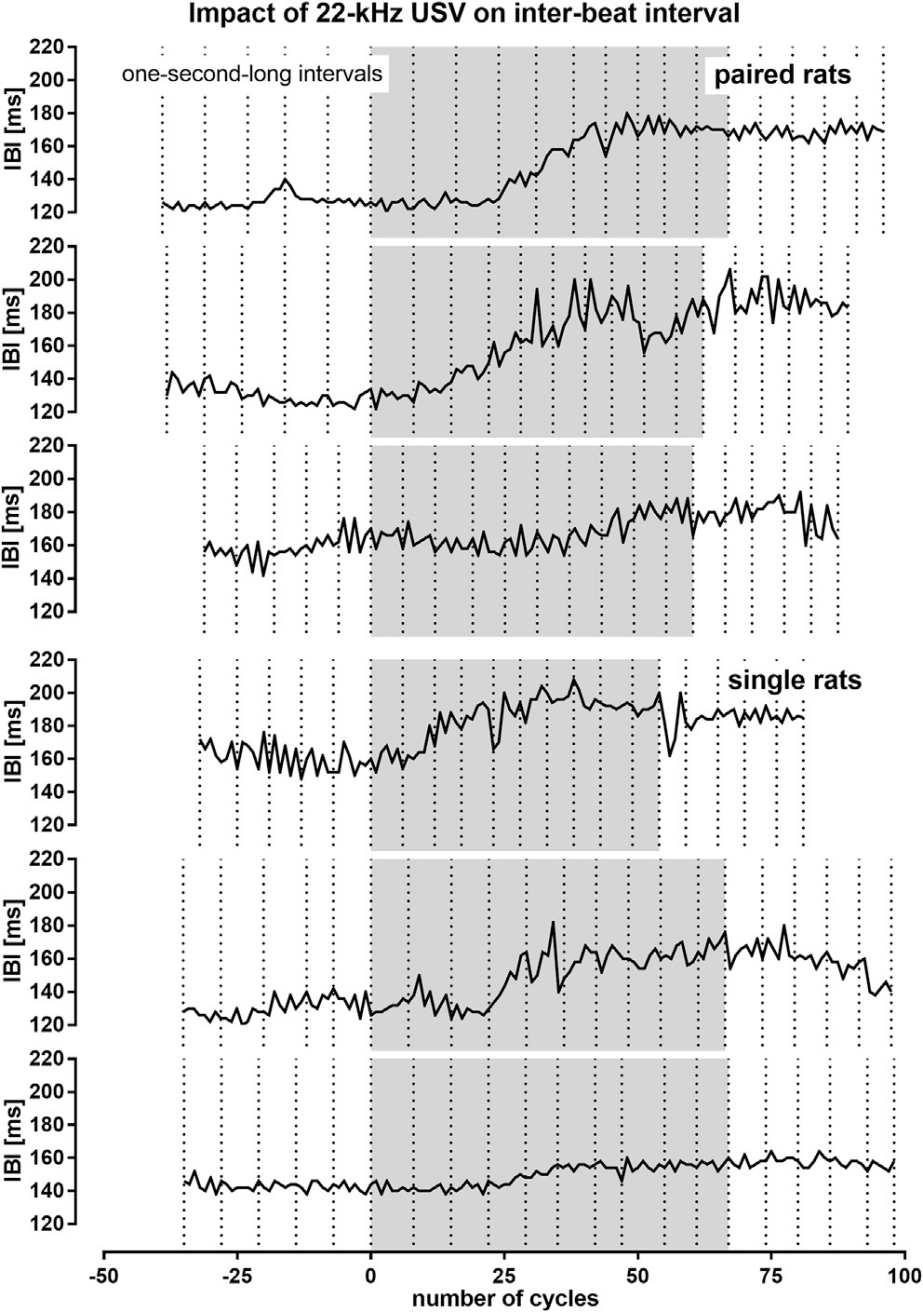
Impact of 22-kHz USV Playback on Inter-Beat Interval (IBI) of Paired (Top Three) and Single (Bottom Three) Randomly Selected Rats That Experienced 22-kHz USV Playback as their First Playback (Comp. Figure 1) Gray sections correspond to the 10-s-long ultrasonic presentation. Dotted vertical lines indicate 1-s-long intervals. In general, 22-kHz USV presentation evoked increase in IBI (i.e. HR decrease); the increase process lasted approx. 2–5 s in each rat.

Finally, we also employed time-resolved analysis of several individual rats exposed to 22-kHz USV playback to determine the duration of HR drop by measuring the heart's inter-beat intervals. We found that the HR decrease takes indeed several seconds, i.e. 2–5 s in investigated animals (Figure 5).

### HR Levels Were Different during and following 50-kHz versus 22-kHz Playback

Fifty-kHz playback resulted in higher HR. It was observed following 50-kHz USV versus 22-kHz USV playback, in the paired (Figure S2B) and single rats (Figure S2D), for 50-kHz tones versus 22-kHz tones in the paired rats (Figure S2F) and in single rats (Figure S2H; see Table S16C for statistics). The differences became more pronounced and significant when both paired and single groups were pooled together for analysis (Figures 4B and 4D; Table S16C) and even more so when the results of USV and tones were pooled together respective to their frequency band for analysis, e.g. 50-kHz USV and tones constituted 50-kHz sounds. Then HR changes in response to the 50 kHz versus 22 kHz sounds differed throughout 0–160 s time-intervals (Figure 4F; Table S16C).

Despite the overall increase in HR in response to 50-kHz sounds, playback of these signals produced short HR drops observed as a difference between −10 s and 0 s time-intervals (Figures 3B and 3D). This initial short HR drop was significant in the paired (p < 0.05) and all rats (p < 0.05, Wilcoxon, Table S11) as well as in all rats with 50-kHz playbacks sounds (USV and tones pooled; p < 0.01, Figure 4F; Table S14C).

### Both 50- and 22-kHz Sounds Evoked an Ultrasonic Response

All ultrasounds presented impacted the number of USV emitted by the rats. These numbers changed in all groups analyzed, especially after the 50-kHz playback in the paired, single (separately, Figures 3B and 3D), and paired and single rats pooled together (Figures 4B and 4D) when presented with 50-kHz USV or 50-kHz tones, as revealed by the analysis of repeated measures (Table S7D, Friedman), i.e. p < 0.001 for all groups at all investigated time-intervals which included 0 s playback interval (i.e. −120 to 180 s, −60 to 60 s, −10 to 10 s) but not during the control time intervals. There was an increase in the number of USV between −10 s time-interval and time-intervals that followed (Table S12).

Similarly, there was also a response following 22-kHz playback (Figures 3F, 3H, 4B, and 4D), e.g. for the paired and single rats analyzed together—for −10 s to 10 s interval after presentation of 22-kHz USV (p < 0.01) and 22-kHz tones (Table S7D, p < 0.05; Friedman) and significant differences between −10 s time-interval and the intervals after 22-kHz USV or tone playback (Table S12).

The majority of calls emitted by the rats during and after the playback, i.e. at 0–120 s time-intervals, were appetitive 50-kHz USV. Notably, it was observed for both 50- and 22-kHz playback, for natural and artificial sounds alike (Table 1). However, 50-kHz USV, emitted by the rats during and after 22-kHz playback, had shorter duration than those emitted during and following 50-kHz playback, which was significant for both USV (p < 0.01) and tones playback (p < 0.01, Wilcoxon), with paired and single rats analyzed together. Please note that a small number of aversive long 22-kHz USV were emitted only following 22-kHz playback, i.e. total of 5 calls in 3 rats following 22-kHz USV and 2 calls in 2 rats following 22-kHz tones.

These changes in the number of USV that were evoked by 50-kHz versus 22-kHz sounds were even more pronounced and significant when the data from paired and single animals as well as USV and tones from respective frequency bands were pooled together into larger groups for analysis (Table S8D; Figure 4F) with prolonged increases in the number of emitted USV as compared with −10 s time-intervals (Table S14D).

### Rats Vocalized More Often during and following 50-kHz Playback Than 22-kHz Playback

Exposition to 50-kHz playback resulted in a dramatic increase in the number of USV emitted (Figures 3B and 3D), whereas the increase was modest during and after 22-kHz sounds presentation (Figures 3F and 3H). When the values of USV emissions following 50- versus 22-kHz playback were compared (Table S16), there was a clear and prolonged difference, which increased in duration and significance as we analyzed more cases together (Figures S2B, S2D, S2F, and S2H versus Figures 4B, 4D, and 4F). As already mentioned, the single rats tended to vocalize more often than the paired ones.

### Natural and Artificial Ultrasounds Produced Similar Results, but Still, Some Differences Stood Out

In general, all the investigated responses to natural versus artificial ultrasounds of the same frequency band were nearly identical and subtle differences were rarely significant when the groups were analyzed separately (Table S17). However, natural calls, as expected, had stronger effects than artificial calls in several instances. The following results are most visible in single rats and pooled groups (paired and single rats together; Figures S3 and S4; Table S17): natural 22-kHz USV reduced distance traveled by single (p < 0.05) and all rats (p < 0.01, Wilcoxon), at 10 s time-interval, more than 22-kHz tone playback (Figures S3E, S3G, and S4C); natural 22-kHz USV reduced HR in single (p < 0.01) and all rats (p < 0.01, Wilcoxon), visible at 0 s time-interval, more than 22-kHz tone playback (Figures S3F, S3H, and S4D); natural 50-kHz USV evoked more USV emissions in response, visible at 10–80 s time-intervals (in most cases significant, Wilcoxon), than 50-kHz artificial tone playback (Figures S3B, S3D, and S4B); and natural 22-kHz USV evoked more USV in response, visible at 50 s (p < 0.05 for single, p < 0.01 for all rats) and 70 s time-intervals (p < 0.05 for all rats, Wilcoxon), than 22-kHz tone playback (Figures S3F, S3H, and S4D).

### There Were Correlations between the Distance Traveled, Number of Vocalizations, and Heart Rate Changes

Next, we investigated the correlations between selected categories of HR levels, USV emissions, distance traveled, and time spent near the speaker (Data S1). Specifically, for the 50-kHz playback, we have correlated **HR** levels at −10, 0, 10, 20, 30 s time-intervals as well as average HR at 0–20 s, 10–30 s, 10–60 s, and 10–120 s (9 categories), with the numbers of **USV** emitted at 0, 10, 20 s intervals, 0–20 s, 0–50 s, and 0–110 s sums of USV as well as differences between the number of USV at −10 s time-interval and 0, 10, 20 s, and 0–10 s averages (10 categories); with **distance** traveled at −10, 0 and 10 s time-intervals, and difference in distance between 0 s versus −10 s and versus 10 s intervals, as well as total distances traveled during 0–20 s and 10–30 s intervals (7 categories); and with **time** spent in the speaker's half of the cage at −10, 0, 10 s, average time spent at 0–20 s and 10–30 s time-intervals, and difference in time between 10 s versus 0 s and −10 s intervals (7 categories). Similarly, for 22-kHz playback, we aligned 9 HR-level categories, 10 number-of-USV-emission categories, 7 distance-traveled categories, and 7 time-spent categories.

**Figure 6:**
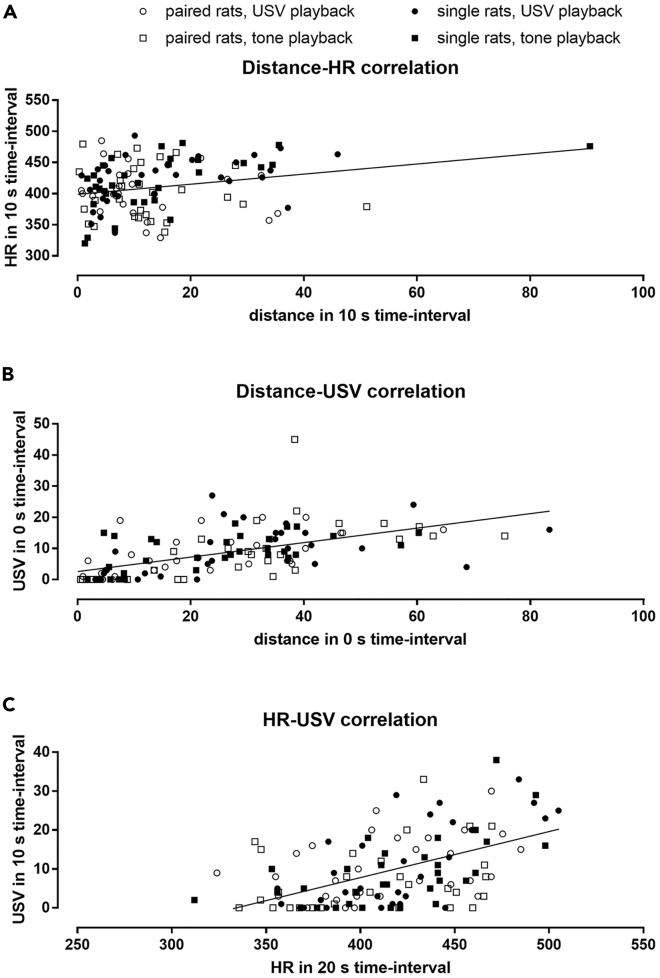
Correlations Obtained for Variables Measured in Response to 50-kHz Playback The correlations are: (A) between distance traveled and heart rate (HR) during 10 s time-interval, (B) distance and number of emitted USV during 0 s interval, and (C) between HR at 20 s and number of USV at 10 s intervals. Correlations were calculated for paired (white marks) and single (black marks) rats exposed to USV (circles) or tones (squares). Straight lines represent linear regressions calculated for all four groups of results presented. For more correlations and p values, see Data S1.

In general, correlations for 50-kHz playbacks were more pronounced than those for 22-kHz ones. Correlations observed in response to 22-kHz sounds were fewer and appeared random (Data S1A and B). There were no strong correlations for the time spent on the speaker's side of the cage for both 50-kHz and 22-kHz playbacks. The observed correlations for 50-kHz playback were especially pronounced in the single rats exposed to natural USV, where we observed 28 significant (p < 0.05, Spearman) distance-HR correlations (of 63 investigated), 47 distance-USV correlations (of 70 total), and 78 HR-USV correlations (of 90 total). At the same time, these numbers were lower for the paired rats exposed to 50-kHz USV (6, 33, 63), single rats exposed to 50-kHz tones (19, 12, 76), and paired rats exposed to 50-kHz tones (9, 49, 0), respectively. The investigated correlations turned out to be more significant when the results of the single and paired rats and USV- and tone-playbacks were pooled together (Figure 6; Data S1).

The most striking and consistent correlation was between distance traveled and the number of emitted USV at 0 s time-interval (Figure 6B; Videos S1 and S2), which was observed in the single rats exposed to 50-kHz USV (0.68, p < 10^−4^) or 50-kHz tones (0.51, p < 0.01) and paired rats exposed to 50-kHz USV (0.65, p < 0.001) or tones (0.77, p < 10^−5^; all Spearman; Data S1A), as well as during 22-kHz playback (Data S1B). There were also distance-HR correlations, e.g. during 10 s time-interval (Figure 6A), which was significant only for the single rats exposed to 50-kHz USV (0.46, p < 0.01) and tones (0.59, p < 0.001; Spearman). Finally, there were numerous HR-USV correlations, e.g. HR levels at 20 s time-interval correlated with the numbers of USV emitted during 10 s interval (Figure 6C), which was observed in the single rats exposed to USV (0.64, p < 0.001) and tones (0.58, p < 0.001) as well as in the paired rats exposed to USV (0.40, p < 0.05; Spearman). The three correlations (Figure 6) were more significant, when the results from both single and paired rats as well as USV and tone playback were pooled for analysis, 0.66, p < 10^−14^ (Figure 6B); 0.40, p < 10^−4^ (Figure 6A); 0.49, p < 10^−6^ (Figure 6C); Spearman.

## Discussion

### In Our Behavioral Model, Rats Vocalize when Exposed to Ultrasonic Playback

We developed an assay of bidirectional communication in rats by exposing individual rats in familiar conditions to ultrasonic playbacks. Rats vocalized copiously, up to >60 calls per minute (Figures 3B and 3D), mostly within the 50-kHz range (Table 1). In similar playback setups, rats vocalized mainly in the 22-kHz range with only 0.2–3.0 50-kHz calls per minute (Wohr and Schwarting, 2007, 2009; Sadananda et al., 2008; Seffer et al., 2015; Berg et al., 2018) or 3.0–4.0 USV of unknown frequency per minute in a mating context (White and Barfield, 1989; White et al., 1993). The observed differences are likely due to steps taken to reduce stress levels, e.g. more handling sessions, longer room habituations, shorter playbacks, home-like-cages (versus elevated maze). We observed more USV during and after 50-kHz versus 22-kHz playback (Figures 4B, 4D, 4F, S2B, S2D, S2F, and S2H; Table S16D) and more USV after hearing natural versus artificially-generated ultrasounds (Figures S3B, S3D, S3F, S3H, S4B, and S4D; Table S17D). The proposed behavioral model can serve to study experimental models of brain diseases (Simola and Granon, 2019) and USV-based communication allowing manipulations of various experimental conditions including, but not limited to, rat (paired versus single, familiar versus unfamiliar), cage (e.g., with conspecific's scent), and playback (flat versus trill).

### HR Increased after 50-kHz Playback, Decreased after 22-kHz Playback

We observed a striking difference in HR levels following 50-kHz versus 22-kHz playback, which lasted for almost 3 min (Figure 4F; Table S16C). To the best of our knowledge, HR changes following rats' exposure to different USV were not described before despite some attempts (Demaestri et al., 2019).

It has been demonstrated that rat 50-kHz USV signal emitter's appetitive state, whereas 22-kHz calls signal an aversive state (Wohr and Schwarting, 2007; Brudzynski, 2009, 2015). Hence, presentation of species-typical signals informing about two opposite emotional states could in turn induce relevant emotional arousal in the receivers. Such scenario is strengthened by the observed pattern of limbic and cortical structures activated by aversive versus appetitive calls. Playback of 50 kHz calls activated predominantly frontal and motor cortices and nucleus accumbens, whereas replay of 22 kHz calls activated perirhinal cortex, basolateral amygdala, and periaqueductal gray (Sadananda et al., 2008; Brudzynski, 2013).

Notably, the nucleus accumbens mediates appetitive behavior and is critically modulated by dopaminergic afferents that are known to encode the value of reward. It was demonstrated that playback of 50-kHz USV leads to phasic and rapid dopamine release in the nucleus accumbens, which was positively correlated with social approach behavior (Willuhn et al., 2014). In contrast, the periaqueductal gray, with its several subdivisions, is regarded as a final common path for defensive responses and was shown to mediate bradycardia and behavioral inhibition. For example, 22-kHz playback induced locomotor hypoactivity and freezing responses in rats, which were paralleled by activation in the ventral region of the caudal periaqueductal gray (Vianna and Brandao, 2003). Also, white noise induced freezing behavior, which was mediated by activation of lateral/ventrolateral periaqueductal gray and evoked HR decrease through parasympathetic outflow (Koba et al., 2016). The two types of calls are therefore processed in distinct neuroanatomical regions, which in turn initiate reward versus defense-related neurotransmission and behavioral responses.

Indeed, reception of USV was shown to incite negative state and to activate fight/flight/freeze system, in response to 22-kHz calls, with avoidance and behavioral inhibition or to incite positive arousal, in response to 50-kHz calls, with approach behavior and self-administration of these USV (Wohr and Schwarting, 2007; Burgdorf et al., 2008; Seffer et al., 2014). We observed such positive arousal of the receivers in the case of 50-kHz playback, since these rats for example emitted abundant amount of 50-kHz USV in response. We also observed effects of possible negative arousal in the receivers of 22-kHz playback, e.g. decreased locomotor activity. Therefore, we provide the example of decoding different vocal signals and initiating relevant and dissimilar physiological changes in conspecifics *via* emotional communication in a process termed before as ethotransmission (Brudzynski, 2013).

Analogous results were seen in infant chimpanzees that responded with cardiac acceleration in response to conspecific laughter, whereas showed deceleratory cardiac responses to conspecific screams (Berntson et al., 1989). Also, humans showed strong and prolonged cardiac deceleration to negatively-associated synthesized spoken words and unpleasant pictures, whereas exposure to pleasant pictures resulted in HR increase (Bradley et al., 2001; Bradley, 2009; Ilves and Surakka, 2012), although the latter effect was not always observed (Bradley et al., 2001).

Notably, HR changes in animals witnessing conspecific distress were also investigated in behavioral models of empathy. HR of mice observing other mice experiencing fear increased initially but decelerated below starting levels with repeated exposures (Chen et al., 2009). Rats exposed to a socially stressed cage-mate showed HR increase after the reunion, which lasted around 10 min and habituated over subsequent exposures (Carnevali et al., 2017). Correspondingly, in human children, HR is augmented in response to others' fear, sadness, anger, and happiness/surprise (Anastassiou-Hadjicharalambous and Warden, 2008). These observations are apparently contradictory to our results, as HR decreased during and following playback of 22-kHz aversive calls in our rats. However, these rats did not have direct contact with their conspecifics during ultrasonic playback. Most likely, mammals display different HR changes when in the physical presence of their conspecific emitting emotionally charged signals, e.g. USV, versus when only exposed to isolated stimuli, e.g. pictures, words, or recorded USV.

In conclusion, we observed a striking difference in opposite HR level shifts in response to 50-kHz versus 22-kHz playback, which are known to function as species-typical signals of opposite affective states, i.e. appetitive versus aversive, respectively. The signals were therefore decoded to initiate relevant and contrasting physiological changes.

### Locomotor Activity Increased during 50-kHz Sounds, Decreased after 22-kHz Playback

The increase in locomotor activity was observed only during the 50-kHz exposure, whereas the 22-kHz playback, more so the natural playback, evoked a 30-s-long decrease only after and not during the playback (Figures 3A, 3C, 3E, and 3G; Table S9). Fifty-kHz calls have been shown before to induce activity and approach, whereas 22-kHz calls led to behavioral inhibition (Wohr and Schwarting, 2007; Burman et al., 2008; Seffer et al., 2014; Snoeren and Agmo, 2014; Davidson and Hurst, 2019; Schonfeld et al., 2020; see, however, Endres et al., 2007). As described earlier, such inhibition was shown to be mediated by the periaqueductal gray upon hearing aversive sounds (e.g. Vianna and Brandao, 2003).

As others before, we also observed the rats' locomotor activity to be stable during 22-kHz playback and to decrease immediately at the end of the stimuli presentation (Brudzynski and Chiu, 1995). Our rats spent more time in the speaker's half of the cage following 50-kHz versus 22-kHz playback (Figures 4A, 4C, and 4E; Table S15B). It has previously been shown that exposure to 50-kHz USV caused rats to spend more time in maze arms proximal to the speaker (Berg et al., 2018).

### Single Rats Responded Differently Than Paired Ones

Vocal communication is a feature of rats' interactions (Brudzynski, 2009); the influence of the social context on USV emission and perception remains mostly unknown. We used young adult rats, 7 weeks old, i.e. after rough-and-tumble play period (Panksepp, 1981) and housed them in pairs or singly for 32 days and tested them at 12 weeks of age. Single animals vocalized more when placed in an empty cage (Figures 2G, 2H, S1C, and S1D; Table S6) during the 10-min silence period (Table S2D) and during the playback session (Tables S12 and S14D). Other studies have demonstrated that post-weaned singly housed rats vocalized more than group-housed rats during rough-and-tumble play (Knutson et al., 1998) and tickling (Panksepp and Burgdorf, 2000; Burgdorf and Panksepp, 2001).

The single rats displayed higher HR throughout the experiment compared with the paired ones (Figures 2E, 2F, 3B, 3D, 3F, 3H, S1A, and S1B; Tables S2C, S5, S11, and S14C). Similarly, group-housed rats and prairie voles displayed lower HR than single-housed ones (Sharp et al., 2002; Grippo et al., 2007), even more so when their home-cages were kept in a room with only single-housed conspecifics (Azar et al., 2011), as is our case. HR was the highest at the beginning and then declined throughout the session in agreement with previous studies (e.g. Richardson et al., 1988).

We also observed that single rats showed a tendency of increased locomotor activity (Figures 2A, 2B, 3A, 3C, 3E, and 3G; Tables S2A, S3, S9, and S13A). There are inconsistencies in previous findings such that some studies show that housing in isolation reduced exploration in novel situations, whereas others described more activity. The discrepancy can be due to a multitude of factors, such as age and stress levels (Dalrymple-Alford and Benton, 1981a; Arakawa, 2018). Isolation during juvenile postnatal days 26–40 (PND26-40) suppressed exploration, whereas isolation at postpuberty (PND51-65) had no effect, and isolation at adulthood (PND114-130) facilitated open-field exploration (Arakawa, 2003, 2005). Our single rats were isolated at PND49-82; therefore, some increase in activity is expected. Stress has also been shown to reduce the locomotor activity of isolated rats (Dalrymple-Alford and Benton, 1981a; Arakawa, 2018). Stress can be lowered by repeated exposure and acclimatizing to testing conditions—as in our case—which resulted in more active singly housed rats compared with the group-housed (Dalrymple-Alford and Benton, 1981a, b; Arakawa, 2003, 2018).

In addition, the single rats spent more time in the speaker's half of the cage during playback sessions than the paired rats (Figures 4A, 4C, and 4E; Tables S10 and S13B), an equivalent of approach behavior described before (Wohr and Schwarting, 2007). It was demonstrated that nonsocially housed male rats are more sociable and have higher social novelty preferences than socially housed counterparts (Templer et al., 2018). Therefore, our single rats were old enough, with alleviated stress levels and socially motivated to demonstrate increased locomotor activity and approach behavior, higher HR, and more ultrasonic emissions in response to playback compared with the paired rats.

### Natural and Artificial Ultrasounds Produced Similar Results; Some Differences Stood out

In our hands, natural 22-kHz USV reduced locomotor activity and HR more than the tone playback version. Also, natural 50-kHz and 22-kHz USV evoked more vocalizations than respective tone presentations. Others have demonstrated playback of natural versus artificial 50-kHz sounds to elicit similar social approaches in adult rats. In contrast, only natural 50-kHz USV playback led to a substantial increase in locomotor activity in juvenile rats. Interestingly, in these experiments, natural 50-kHz calls tend to elicit more USV, but the difference was not significant (Figure 8 in Wohr and Schwarting, 2007). Finally, c-Fos studies suggest different neuronal pathways to process natural versus artificial 22-kHz playbacks (Ouda et al., 2016).

### Vocalizations, HR, and Locomotor Activity are Co-regulated

The changes in HR, USV, and locomotor activity all correlated in response to 50-kHz playback especially. Such correlations were observed before. Regarding **HR level–USV emission correlation**, similar one was documented in voles between HR changes and frequency of emitted USV (Stewart et al., 2015). Such correlation was expected since USV-linked respiration and HR are tightly regulated, e.g. with common regulatory brain structures, e.g. nucleus ambiguus and afferent feedback (Porges, 2007). Also, common signaling pathways (e.g. vagus nerve) and cerebral centers (e.g. the nucleus of the solitary tract and nucleus ambiguus) regulate HR levels as well as laryngeal and pharyngeal muscles' tension (Stewart et al., 2015). We obtained a highly significant correlation between the number of USV emitted immediately after 50-kHz playback, at 10 s time-interval, and HR of the next interval, at 20 s (Figure 6C), i.e. the most pronounced correlation was for USV emissions and subsequent HR levels, i.e. with USV emission—HR time sequence.

We also observed a **distance traveled–HR level correlation**. Several vagal reflexes exist that produce rapid HR decelerations, e.g. in response to movement, intention to move, posture shifts, and breaths (Porges, 2007); these reflexes influence movement itself. Also, the vagus nerve tone affects many animal behaviors (Stern, 1997; Look at Figure 1 in Porges, 2007); therefore, changes in HR may reflect or be caused by changes in locomotor activity. We, however, observed HR to drop before the decrease in the activity following the 22-kHz playback (Figure 4). Also, the distance-USV correlations were much more pronounced than distance-HR correlations.

Finally, **distance traveled–USV emission correlations** were found to be the strongest (Figure 6B; Data S1). This supports previous studies of rats synchronizing their locomotion and USV on a subsecond timescale, which implicates a role of mesolimbic dopaminergic pathway and of the basal ganglia (Laplagne and Elias Costa, 2016). Overall, it would be difficult to separate all effects concerning locomotor activity, HR, and USV emission, because they, at least in part, originate from the same vagal path. Their coordinated regulation can be explained by the polyvagal theory (Porges, 1995, 2007, 2009).

## Conclusion

**Figure 7:**
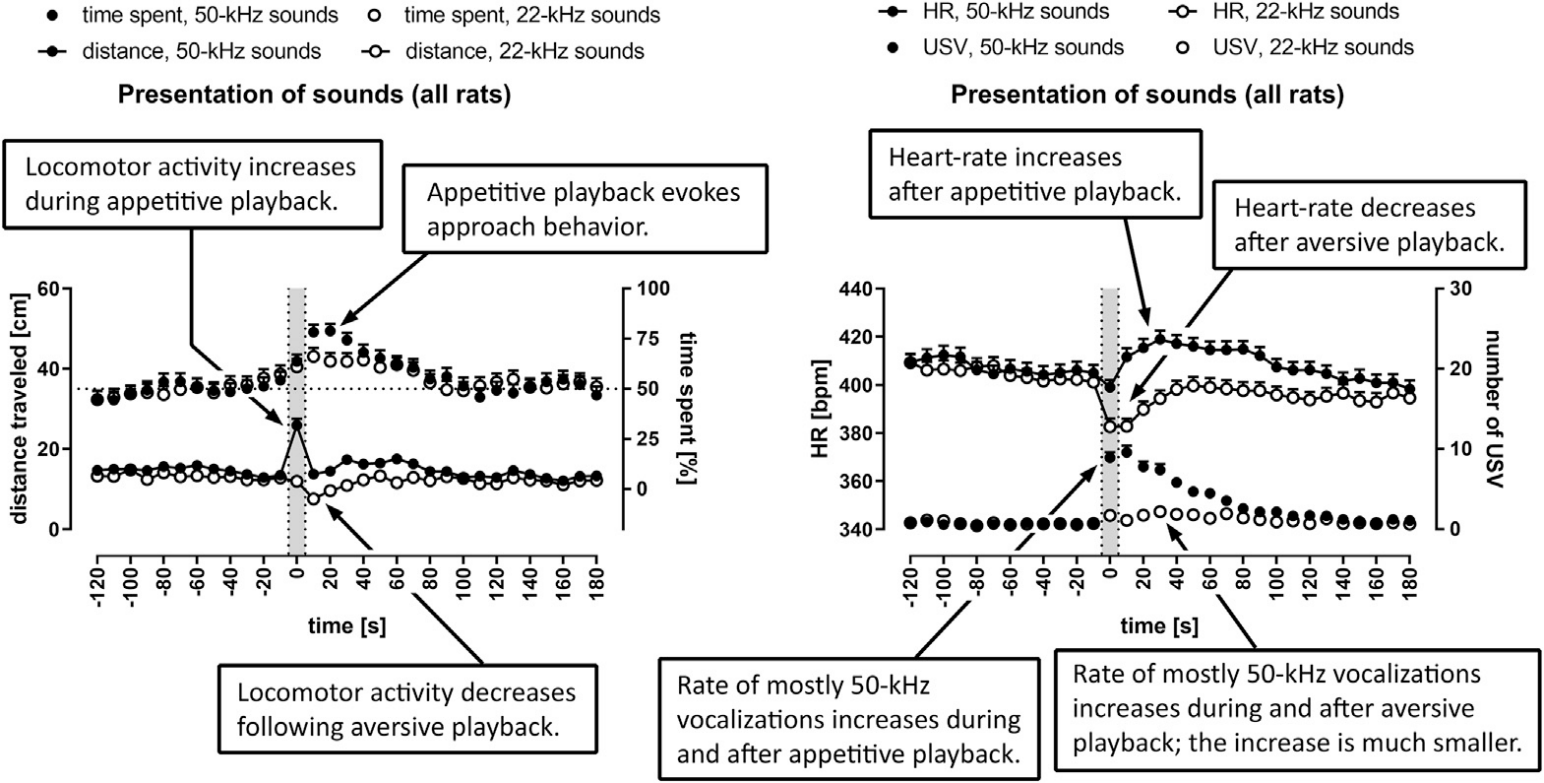
Key Results of the Present Work Main findings can be seen on the right panel with heart rate increasing or decreasing following 50-kHz versus 22-kHz playback, respectively, as well as increased vocalization following ultrasonic playback. Paired and single rats as well as responses to respective USV and tones (sounds) were pooled together for analysis (comp. Figures 4E and 4F).

We describe a rat behavioral model of vocal communication to study exchange of ultrasonic calls by measuring and analyzing numerous USV produced in response to appetitive and, to a lesser extent, to aversive playback. The number of USV emitted correlated with increased locomotor activity and HR changes. Fifty-kHz playback evoked vocalizations and increased locomotor activity, and approach behavior, whereas 22-kHz playback led to reduction in locomotor activity. Most notably however, HR gradually increased following 50-kHz playback, whereas sharply decreased following 22-kHz exposure. Main results are also summarized in Figure 7.

Fifty-kHz and 22-kHz calls signal rat contrasting emotional states, and we demonstrate that these USV are able to evoke appetitive or aversive states in signals' receivers. Therefore, we also introduce objective physiological and behavioral signatures of affective conditions, filling the need of objective measures of emotional states, and further confirm the biological role of vocal, emotional, valence-depended communication.

### Limitations of the Study

There are some methodological weaknesses in our work. Fifty-kHz signal presented was a specific, selected group of USV. A different combination of USV may give different results, e.g. more or less USV in response. A more detailed and systematic presentation of particular types of USV and their combinations would be more precise and will be the subject of further studies. Moreover, each rat was presented with all four playback signals (Figures 1B–1E) in counterbalanced order; therefore, the experimental design presents both an element of habituation as well as additional variability in data obtained. The results could be, potentially, less variable, but would require more animals. Also, although the implantation of radiotelemetric transmitters is a major surgical procedure, which could have some influence on the behavior and physiology (i.e. HR) of the animals, they are nevertheless regarded as the state-of-the-art method for monitoring physiologic functions in awake and freely moving rats while minimizing stress-associated artifacts (Braga and Prabhakar, 2009). In addition, HR was measured and averaged over 10-s-long intervals; although a standard procedure, a more detailed study is being planned. Finally, this experiment was conducted on laboratory rats, and it should be noted that in the wild, rats may behave differently and display a wider range of calls (comp. Kalcounis-Rueppell et al., 2010).

### Resource Availability

#### Lead Contact

For correspondence, contact Robert K. Filipkowski (rfilipkowski@imdik.pan.pl)

#### Materials Availability

This study did not generate new materials.

#### Data and Code Availability

Raw data, analyzed herein, have been deposited to Mendeley Data at http://dx.doi.org/10.17632/b4m46fwytg.1.

## Methods

All methods can be found in the accompanying Transparent Methods supplemental file.

## Supplemental Information

Document S1. Transparent Methods, Figures S1–S4, and Tables S1–S17

Data S1. Correlations between Selected Categories of Heart Rate, USV, Distance and Time in Paired, Single and Pooled Groups of Rats, Related to Figure 6 Spearman's rho and color-coded p values are given.
