# Supplementary material for: Playback of Alarm and Appetitive Calls Differentially Impacts Vocal, Heart-Rate, and Motor Response in Rats
Source: iScience. 2020 Sep 19;23(10):101577. doi: 10.1016/j.isci.2020.101577 (PMC7553343; doi:10.1016/j.isci.2020.101577)
Supplement: Document S1. Transparent Methods, Figures S1–S4, and Tables S1–S17 [file mmc1.pdf]

iScience, Volume *23*

Supplemental Information

Playback of Alarm and Appetitive Calls Differentially Impacts Vocal, Heart-Rate, and Motor Response in Rats

Krzysztof H. Olszyński, Rafał Polowy, Monika Małż, Paweł M. Boguszewski, and Robert K. Filipkowski

Supplemental Materials

Transparent Methods

Animals and housing

All experiments were approved by the Fourth Local Ethical Committee in Warsaw. Naïve adult male Wistar rats (7 weeks of age) obtained from The Center for Experimental Medicine of the Medical University of Bialystok, Poland, were kept in two separate rooms. In the first one, all the rats were housed in pairs (n = 30 pairs, "paired rats"); the second room harbored single-housed rats only (n = 31, "single rats"). Standard chow and water were provided *ad libitum*. Both housing rooms had a 12 h light-dark cycle and an ambient temperature of 22–25 °C. All experiments were conducted during the light cycle (9:00–21:00 h). There were 32 days between the arrival and the experiment day. In the first two weeks, the rats were habituated to new facilities. Then all animals (including both rats in each pair) were handled once 2 min per day for 12 days prior to surgery.

Surgical procedures

For one rat from each pair and for every single rat, a radiotelemetric transmitter (HD-S10, Data Sciences International, St. Paul, MN, USA) for cardiovascular studies was disinfected using Cidex® (Johnson&Johnson, New Brunswick, NJ, USA) and implanted under ketamine-xylazine anesthesia. The abdominal region was shaved and disinfected (Octenisept, Schulke, Norderstedt, Germany). A midline incision was performed, the transmitter sensor was implanted into the abdominal aorta by direct puncturing of the vessel and fixing with tissue glue (Histoacryl®, B. Braun, Melsungen, Germany). The transmitter body was placed in the peritoneal cavity and fixed to the abdominal muscle wall. After surgery, the animal was subcutaneously injected with Metacam (0.4 mg/kg; Boehringer Ingelheim, Ingelheim am Rhein, Germany) for analgesia. Post-surgical recovery lasted another week during which the animals were handled and habituated to experimental conditions 4 times.

Experimental design and settings

On the day of the experiment, the animals were 12 weeks of age and had spent 32 days either with a cage-mate (paired rats) or alone (single rats). The animals were transferred into their individual experimental cages, identical to home cages (plastic; 37 x 25 x 16 cm), and transported to the experimental room, which was done during the weekend to minimalize human presence and noises in the animal house, under white light, in the absence of the experimenter and other rats in the room. Acoustic stimuli were presented through an ultrasonic speaker (Vifa, Avisoft Bioacoustics, Berlin, Germany), placed just above the shorter side of the cage, connected to an UltraSoundGate Player 116 (Avisoft Bioacoustics). USV emitted by the rat were recorded by Condenser Microphone (UltraSoundGate CM16/CMPA, Avisoft Bioacoustics) placed 33 cm above the center of the cage floor, 20 cm away from the speaker. In this configuration, calls from the speaker were still visible in the recording (monitoring of playback), but they were distinctively weaker than USV emitted from the cage. Both playback and recording were performed using Avisoft Recorder USGH software (Avisoft Bioacoustics). The locomotor activity of the animal was recorded with a camera (acA1300-60gc, Basler AG, Ahrensburg, Germany) mounted above the cage and EthoVision XT software (version 10, Noldus, Wageningen, Netherlands). Signals from radiotelemetric transmitters were collected by receivers located under the cage floor and then recorded by Dataquest ART 4.36 software (Data Sciences International, St. Paul, MN, USA).

Ultrasonic-playback presentation

Upon placing rat into the experimental cage, a 10 min of silence, i.e. background noise of 20.6 ± 0.2 dB, was followed by four 10-s-long sets of signals, separated by 5-min-long silence intervals (Figure 1). Four set of signals (playbacks) were presented in counterbalanced order to each rat: i. 50-kHz natural calls (referred to as "50-kHz USV"), 84 calls in 3 repeats, of 49.2 to 73.4 kHz frequency and 58.6 ± 0.7 kHz mean peak frequency, 28.4 ± 1.6 ms duration, 31.9 ± 0.6 dB sound pressure, recorded during rats'

social interactions; ii. 50-kHz software-generated tones ("50-kHz tones"), 32.6 ± 0.7 dB; iii. 22-kHz natural calls ("22-kHz USV"), 24 calls in 8 repeats, 21.4–23.0 kHz, 22.1 ± 0.1 kHz, 375.3 ± 21.6 ms, 38.3 ± 1.2 dB, recorded during fear conditioning (Avisoft Bioacoustics [Internet]; c2020 Examples of rat ultrasonic vocalizations (USV), Norwegian Rat (Rattus norvegicus), Wistar albino strain, Males); and iv. 22-kHz software-generated tones ("22-kHz tones"), 43.3 ± 3.0 dB, although the sound playbacks of the same frequency range, e.g. 50-kHz USV and 50-kHz tones, always followed each another. Artificial tones were generated based on natural ones (mean peak frequency, duration, pauses between tones in the set, but with no frequency modulations) using Avisoft SASLab Pro (Avisoft Bioacoustic) (Figure 1). Calls were presented with a sampling rate of 200 kHz in 16-bit format. The sound pressure levels of the background noise and playback signals were assessed in the middle of the test cage's floor, at the height of animals' typical head position, facing the speaker.

Analysis of USV and locomotor activity

Recordings were transferred to SASLab Pro (Avisoft Bioacoustics), and a fast Fourier transform was conducted (512 FFT-length, 100% frame, Hamming window and 75% time window overlap), resulting in high resolution spectrograms (frequency resolution: 391 Hz; time resolution: 0.64 ms). An experienced user counted the number of USV manually. Automated video tracking system (Ethovision, Noldus, Wageningen, The Netherlands) was used to measure total distance traveled (cm), a measure for general locomotor activity, and proximity to speaker, i.e. time spent (%) in the half of the cage closer to the speaker. Center-point of each animal's shape was used as a reference point for measurements of locomotor activity, thus registering only full-body movements, i.e. distance traveled by a given rat.

Statistical analysis

All data were analyzed using Friedman, Wilcoxon, and Mann-Whitney tests with Statistica 7.1 (Stat-Soft) due to prevailing non-parametric distribution of the results; the p values are presented in the tables, with a minimal level of significance of $p < 0.05$. Figures were prepared using GraphPad Prism 7 software and depict mean values with a standard error of the mean (SEM). The data are average values for 10-s-long time-intervals. For additional analysis of the first 10-min data, outliers for USV emissions were determined by Tukey's fences method (Johansen and Christensen, 2018; van der Spoel et al., 2019) and excluded along with non-vocalizers, which reduced the groups to $n = 21$ (paired) and $n = 25$ (single rats). Due to equipment error, we recorded no distance and time data of one of the paired rats (hence $n = 29$). Between-variables co-efficiency was measured with nonparametric Spearman's correlation (rho, ρ). We realize that pooling together of data for analysis, e.g. of responses to 50-kHz USV and 50-kHz tones of the same animals, is not entirely justified; therefore, we report findings from our primary groups, i.e. paired, single, and all rats; while the findings and effects observed in pooled data serve to further support and emphasize the results observed for the primary groups.

Literature cited in Methods

1. Johansen MB, Christensen PA (2018) A simple transformation independent method for outlier definition. Clin Chem Lab Med 56:1524-1532.

2. van der Spoel E, Choi J, Roelfsema F, Cessie SL, van Heemst D, Dekkers OM (2019) Comparing Methods for Measurement Error Detection in Serial 24-h Hormonal Data. J Biol Rhythms 34:347-363.

Supplemental Materials (Figures)

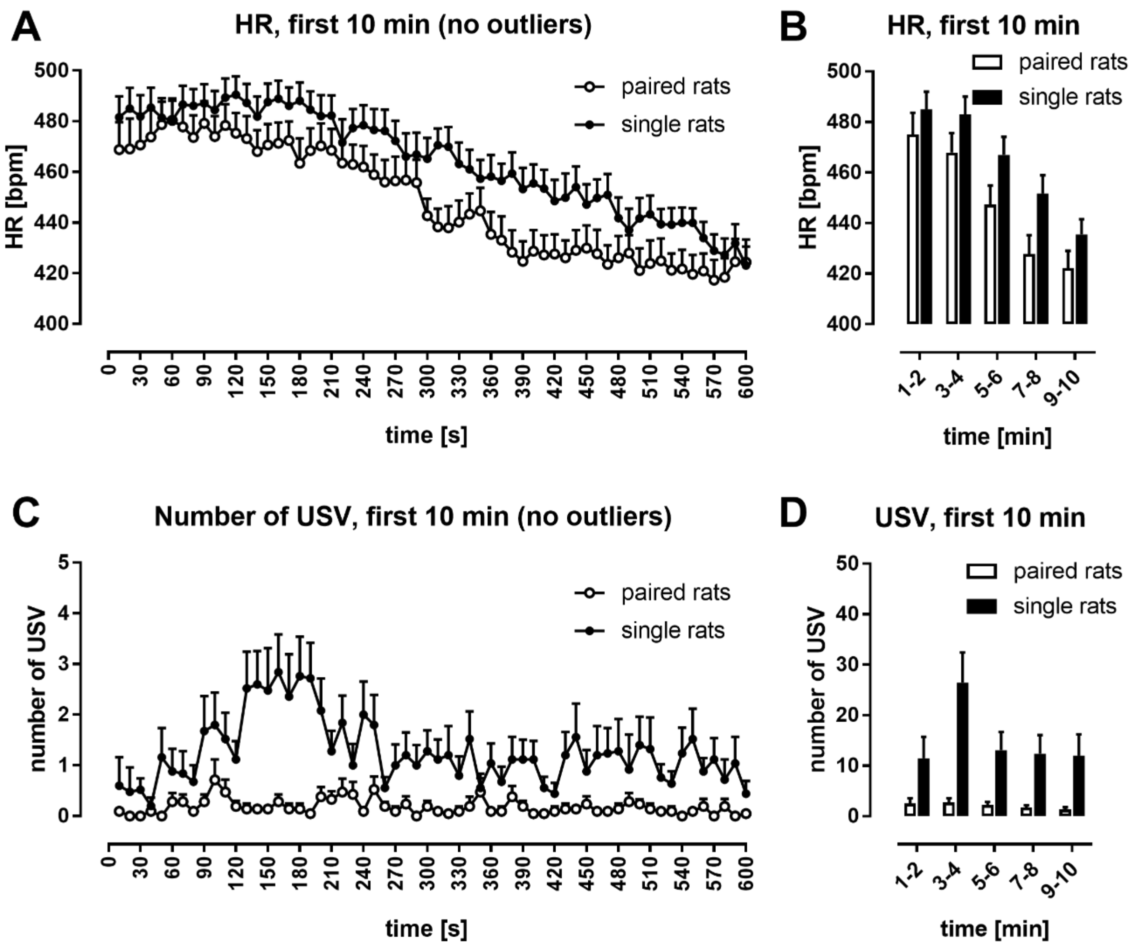

Figure S1. Changes in heart rate (HR) and the number of USV emitted by rats during the first 10 min of silence in the experimental cage calculated after eliminating non-vocalizers and outliers. Related to Figure 2. The figure is a modification of Figure 2*E-H* (paired rats, n = 21; single, n = 25); (**A**, **B**) heart rate (bpm; beats per minute); (**C**, **D**) number of USV. Line charts (**A**, **C**) are presented as means with SEM for 10-s intervals. Bar graphs (**B**, **D**) show means with SEM for 2-min intervals. Values for paired rats are presented as blank dots and bars, for single rats – as solid dots and bars. Single rats had higher HR and vocalized more than paired rats; the differences were strengthened after elimination of outliers; for p values, see Table S1*CD*, Table S2*CD*, Table S5, and Table S6.

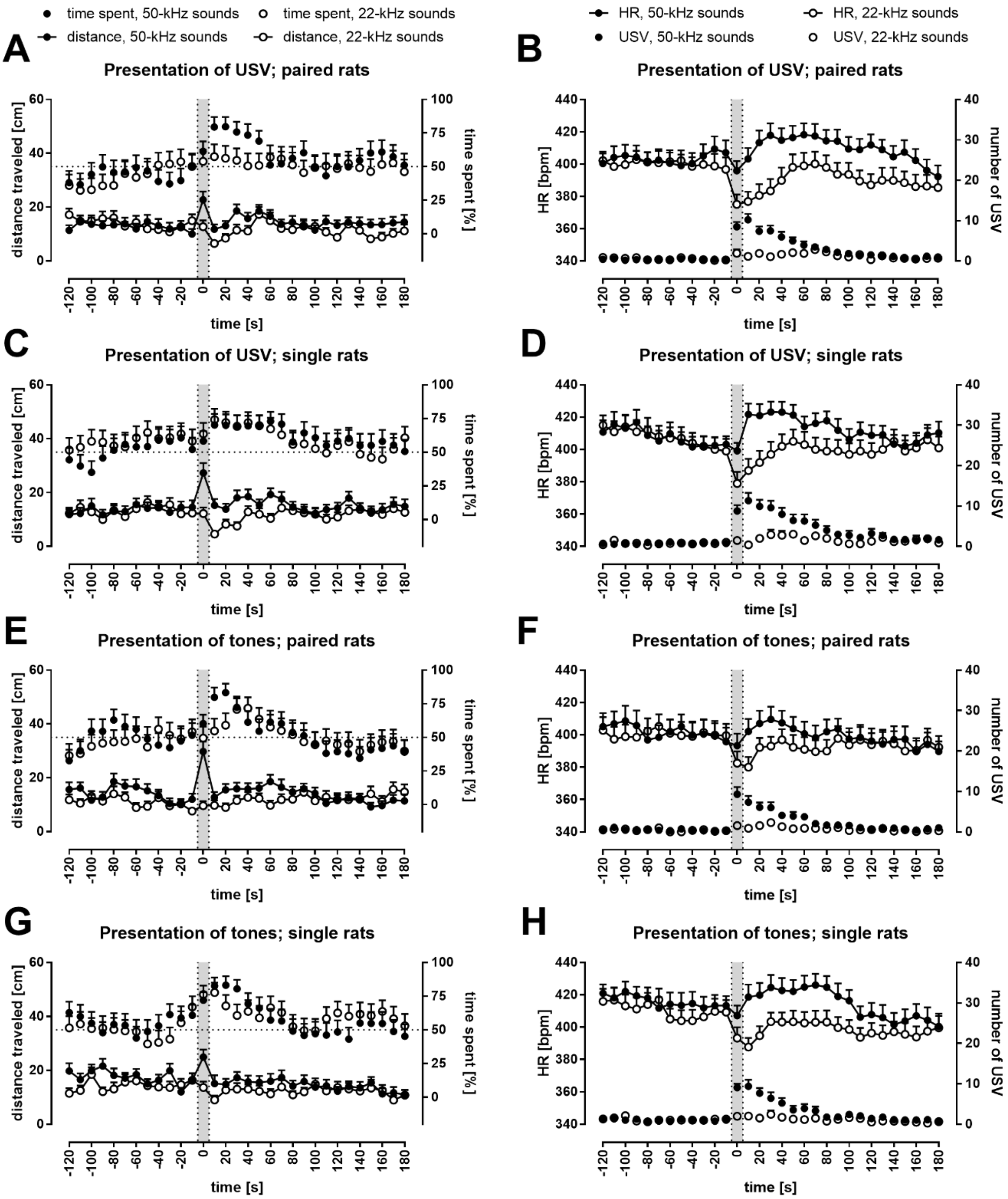

Figure S2. Comparison of responses to 50-kHz vs. 22-kHz ultrasonic playback in paired and single rats. Related to Figure 3. Gray sections correspond to the 10-s-long time-intervals of ultrasonic presentations. Graphs depict responses after exposure of paired rats to USV (**A, B**), single rats to USV (**C, D**), paired rats to artificial tones (**E, F**), single rats to tones (**G, H**). Responses to 50-kHz signals is presented as solid dots, to 22-kHz signals – as blank dots. In the left column (**A, C, E, G**), distance traveled is presented as connected dots (cm, left Y axis), percentage of time spent in the half of the cage with the speaker is shown as not connected dots (%, left Y axis). The dotted horizontal line marks a 50% chance value for time spent in the speaker's half. In the right column (**B, D, F, H**), HR is presented as connected dots (bpm, beats per minute, left Y axis); the number of USV is shown as not connected dots (right Y axis). Each data point is a mean for the 10-s-long time-interval with SEM; for p values, see especially Table S16; paired, n = 29/30; single, n = 31.

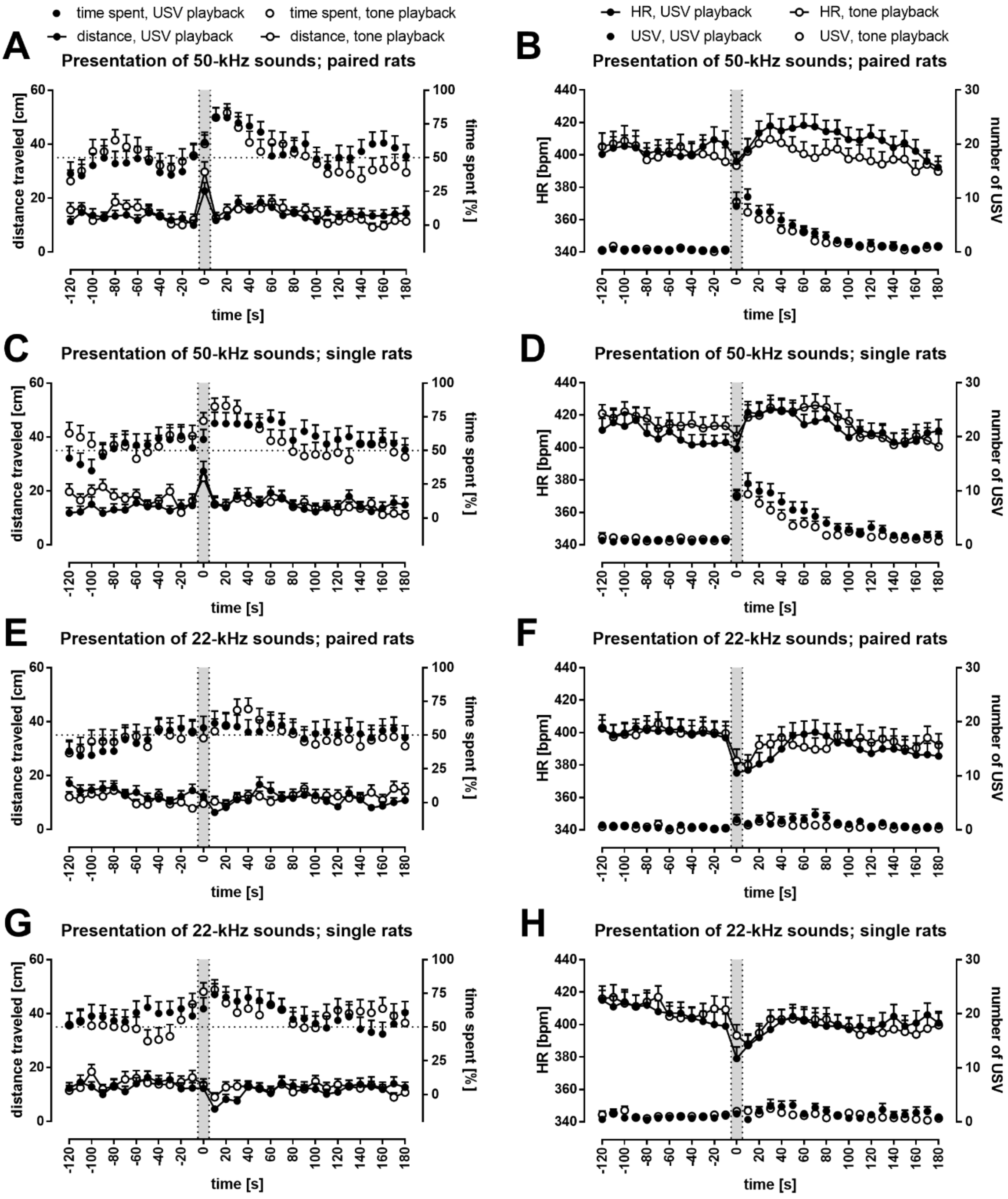

Figure S3. Comparison of responses to playback of natural USV vs. artificial tones in paired and single rats. Related to Figure 3. Gray sections correspond to the 10-s-long time-intervals of ultrasonic playback. Graphs depict responses after exposure of paired rats to 50-kHz sounds (**A**, **B**), single rats to 50-kHz sounds (**C**, **D**), paired rats to 22-kHz sounds (**E**, **F**), single rats to 22-kHz sounds (**G**, **H**). Response to natural USV is presented as solid dots, to artificial tones – as blank dots. (**A**, **C**, **E**, **G**) Distance traveled is presented as connected dots (cm, left Y axis), percentage of time spent in the half of the cage – as not connected dots (%, right Y axis) with dotted horizontal line marking a 50% chance value for time spent. (**B**, **D**, **F**, **H**) HR is presented as connected dots (bpm, beats per minute, left Y axis); the number of USV is shown as not connected dots (right Y axis). Each point is a mean for the 10-s-long time-interval with SEM; for differences in USV vs. tones, see p values in Table S17; paired, n = 29/30; single, n = 31.

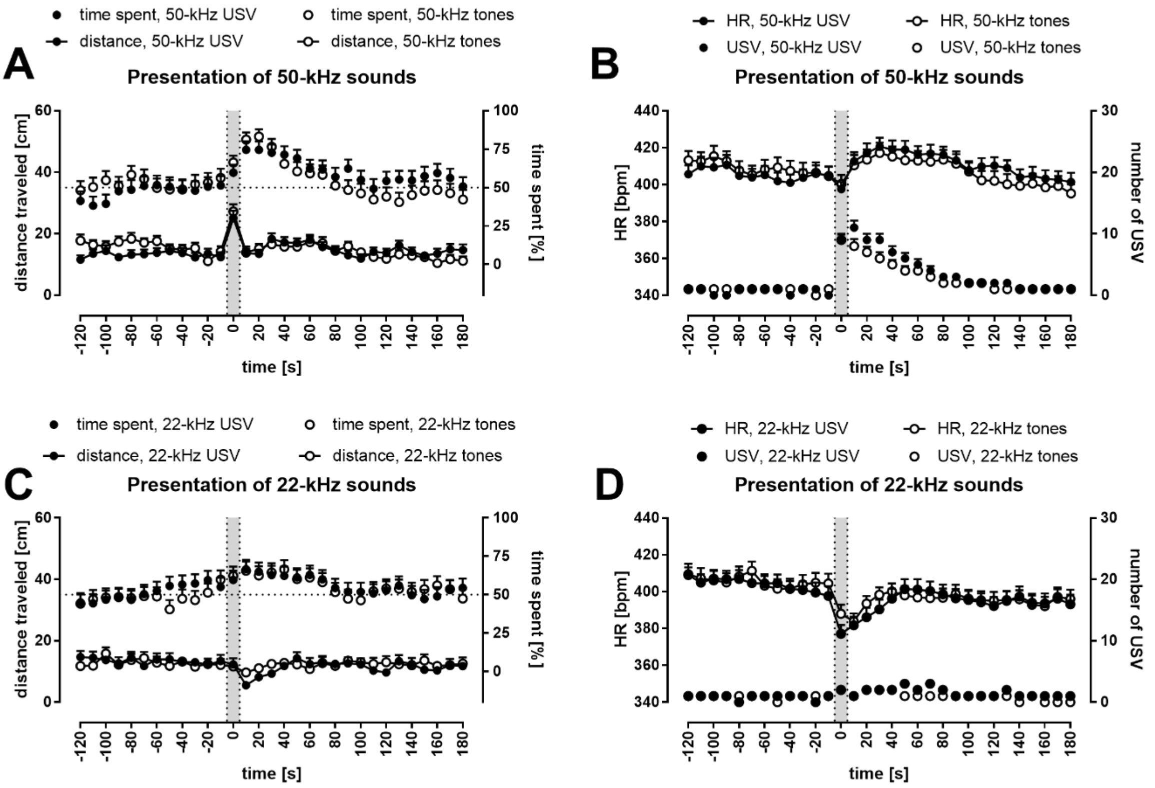

Figure S4. Comparison of rats' responses to natural USV vs. artificial tones with paired and single animals pooled together for analysis. Related to Figures 3 and 4. Gray sections correspond to the 10-s-long time-intervals of ultrasonic presentations. Graphs show responses to playbacks of 50-kHz sounds (**A**, **B**) and 22-kHz sounds (**C**, **D**). Responses to natural USV are presented as solid dots, to artificial tones – as blank dots. (**A**, **C**) Distance covered is shown as connected dots (cm, left Y axis). Percentage of time spent in half of the cage with the speaker is presented as not connected dots (%, right Y axis). (**B**, **D**) HR is shown as connected dots (bpm, beats per minute, left Y axis); the number of USV is shown as not connected dots (right Y axis). Each data point is a mean for the 10-s-long time-interval with SEM. The dotted horizontal line marks a 50% chance value for time spent in the speaker's half of the cage. The most prominent changes were: increased number of USV (**B**) and increased locomotor activity (**A**) during 50-kHz playback, as well as a decrease in HR (**D**) and locomotor activity (**C**) during and/or after 22-kHz sounds presentation. Several differences in responses to USV vs. tones for 50-kHz (**A**, **B**) or 22-kHz signals (**C**, **D**) were observed; for description and p values, see the text and Table S17; n = 60 for **A**, **C**; n = 61 for **B**, **D**.

Supplemental Materials (Tables)

	all animals			no outliers		
interval [s]	**paired**	**single**	**all rats**	**paired**	**single**	**all rats**
**A. distance**	n = 29	n = 31	n = 60	n = 20	n = 25	n = 45
10 – 120	p = 0.70	p < 0.05	p = 0.11	p = 0.71	p < 0.05	p < 0.05
130 – 600	p = 0.28	p = 0.19	p = 0.09	p = 0.17	p = 0.58	p = 0.35
**B. time**	n = 29	n = 31	n = 60	n = 20	n = 25	n = 45
10 – 120	p < 0.05	p < 0.001	p < 0.05	p = 0.31	p = 0.10	p = 0.54
130 – 600	p = 0.10	p = 0.18	p < 0.01	p = 0.50	p = 0.10	p < 0.01
**C. HR**	n = 30	n = 31	n = 61	n = 21	n = 25	n = 46
10 – 120	p = 0.63	p < 0.05	p = 0.22	p = 0.50	p = 0.14	p = 0.51
130 – 600	p < 0.001	p < 0.001	p < 0.001	p < 0.001	p < 0.001	p < 0.001
**D. # of USV**	n = 30	n = 31	n = 61	n = 21	n = 25	n = 46
10 – 120	p = 0.12	p < 0.001	p < 0.001	p < 0.05	p < 0.001	p < 0.001
130 – 600	p = 0.07	p < 0.001	p < 0.001	p = 0.44	p < 0.001	p < 0.001

Table S1. Distance traveled (A), time spent in the speaker's half (B), heart rate (C), and the number of USV (D) analyzed for changes during the first 10 min of silence. Related to Figures 2 and S1. Differences within groups, between different time-points, were evaluated for 10–120 s and 130–600 s time-intervals in paired, single, and all rats as well as in these groups without outliers; significant (< 0.05) or exact p values are given; all Friedman's tests.

	all rats					no outliers				
[min]	**1 – 2**	**3 – 4**	**5 – 6**	**7 – 8**	**9 – 10**	**1 – 2**	**3 – 4**	**5 – 6**	**7 – 8**	**9 – 10**
**A. distance traveled**										
	**paired (n = 29) vs. single (n = 31)**					**paired (n = 20) vs. single (n = 25)**				
interval			+	+	+			+	+	+
**B. time in speaker's half**										
	**paired (n = 29) vs. single (n = 31)**					**paired (n = 20) vs. single (n = 25)**				
interval	+	+			+	+	+			+
**C. HR changes**										
	**paired (n = 30) vs. single (n = 31)**					**paired (n = 21) vs. single (n = 25)**				
interval	+	+	+	p < 0.05+	+	+	+	+	p < 0.05+	+
**D. number of USV**										
	**paired (n = 30) vs. single (n = 31)**					**paired (n = 21) vs. single (n = 25)**				
interval		p < 0.05+	p < 0.05+	+	p < 0.05+	p < 0.01+	p < 0.01+	p < 0.01+	+	p < 0.01+

Table S2. Differences between paired and single rats during the first 10 min of silence. Related to Figures 2 and S1. Results of distance traveled (**A**), time spent in the speaker's half (**B**), heart rate (**C**), and the number of USV (**D**) were analyzed for 2-min-long time-bins in paired, single, and all rats as well as in these groups without outliers. Statistically significant differences between the results of paired vs. single rats are marked with p < 0.05 or < 0.01, all Mann-Whitney tests; (+) indicates a higher value for the single group.

time-interval [s]	all animals (60 rats)			no outliers (45 rats)			all animals	no outliers
	compared to average values across 10 min, p values						paired vs. single p values	
	paired	single	all rats	paired	single	all rats		
10		+			+		+	+
20		0.001	0.005		0.001	0.005		
30		0.002	0.031	+	0.012			
40		0.009			0.009	0.022		
50		+					+	
60				+				
70	+							
80	+			+				
90	+		+	+	+	+		
100		+	+	+	+	+	+	+
110	+		+	+				
120	+		+	+		+		
130		+	+	+	+	+	+	+
140		+	+	+		+	+	
150	+		+	+		+		
160	+	+	+		+	+		+
170	0.037+	+	+	+	+	+		
180	+	+	+		+	+	+	+
190	+	+	+		+		+	+
200	+	+	+	+	+	+	+	+
210	+		+	+		+		
220	+			+				
230	+	+	0.035+	+	+	+		
240	+	+	+	0.033+	+	+	+	
250	+	+	+	+	+	+	+	+
260							+	
270		+	+		0.045+	+	+	+
280	+	+	+		+	+	+	+
290	+		+	+		+		
300	+		+	+	+	+		
310	+	+	+	+	+	+	+	+
320	+	+	+		+	+	+	+
330		+	+		+	+	+	+
340	0.024	+					+	+
350		0.010	0.015	+	0.021			
360		+		+		+	+	
370				+				
380	0.041		0.005				+	+
390	+	+	+	+	+	+		+
400		+			+		+	+
410		+	+	+	+	+	+	+
420	+			+				
430	+	+	+	+	+	+	+	+
440		+	+		+	+	+	+
450				+	+	+	+	+
460				0.030	+		+	+
470		+					+	+
480							+	+
490		0.048						
500	0.039		0.014	0.048		0.014	+	+
510		+			+	+	+	+
520	+		0.026					+
530	+	+	+	+	+	+		+
540		+			+		+	+
550	+	+	+	+	+	+	+	+
560	+	+	+		+	+	+	+
570							+	+
580	0.018	+			+		0.035+	+
590		+			+		+	+
600		+			+		+	+
	47%	57%	50%	50%	55%	52%	36/60 (60%)	35/60 (58%)

Table S3. Analysis of distance traveled during the first 10 min of silence of the playback session. Related to Figures 2 and S1; for paired (n = 29), single (n = 31) and all rats (n = 60), and these groups without outliers (i.e. with n = 20, 25, 45; respectively), divided into 10-s-long intervals. Left part; comparison of given results to average values for entire 10 min; Wilcoxon's tests; (+) denotes locomotor activity higher than average. Right part; significant differences between paired and single groups – for all rats and after eliminating outliers; Mann-Whitney tests; (+) denotes each instance of higher value for single rats. Significant (< 0.05) p values are given.

time-interval [s]	all animals (60 rats)			no outliers (45 rats)			all animals	no outliers
	compared to 50% value, p values						paired vs. single p values	
	paired	single	all rats	paired	single	all rats		
10		0.040+			+	+	0.015+	+
20	+	+	+		+			+
30	+		+	+				
40		+					+	+
50							+	+
60	+		+	+				
70	+		+	+				
80	+		+	+				
90								
100			0.037				+	+
110		+					+	+
120	0.010			0.021			+	+
130							+	+
140								
150		+		+	+	+	+	+
160		+		+	+	+	+	+
170	+	+	0.043+	+	+	+	+	+
180	+	+	+	+	+	+	+	+
190	+	+	+	+	+	+	+	
200				+				
210		+		+	+	+	+	
220		+			+		+	+
230	+	+	+					
240	+	+	+	+	+	+	+	+
250		+			+	+	+	+
260	+	+	+	+	+	+		
270	+	+	+	+	+	+		+
280	+		+	+				
290						0.048		
300			0.018			0.047		
310	+		+	+				
320	+		+	+		+		
330	+	+	+	+	+	+		
340	+	+	0.044+	0.048+	+	0.008+	+	
350	+	+	+	+	+	+	+	+
360	+	+	+	+	+	+	+	+
370				+				
380							+	
390							+	+
400		+	+	+		+	+	
410		+			+		+	+
420				+		+		
430							+	+
440					+		+	+
450	+		+	+	+	+		
460	+		+	+		+		
470	+			+		+		
480	+	+	+	+		+		
490		+			+		+	+
500	0.009	+		0.017	+		0.035+	+
510	0.039						+	+
520							+	+
530								
540	+	+	+	+	+	+		
550	+		+	+		+		
560	+		+					
570								
580		+					+	+
590		+	+		+	+	+	+
600	+	0.040+	0.022+	+	+	+	+	+
	45%	48%	47%	52%	42%	43%	32/60 (53%)	29/60 (48%)

Table S4. Analysis of time spent in the speaker's half during the first 10 min of silence of the playback session. Related to Figures 2 and S1; for paired (n = 29), single (n = 31) and all rats (n = 60), and these groups without outliers (i.e. with n = 20, 25, 45; respectively), divided into 10-s-long intervals. Left part; comparison of the results to 50% chance level; Wilcoxon's tests; (+) denotes residing longer in the speaker's half. Right part; significant differences between paired and single rats – for all subjects and after eliminating outliers; Mann-Whitney tests; (+) denotes each instance of higher value for single rats. Significant (< 0.05) p values are given.

time-interval [s]	all animals (61 rats)			no outliers (46 rats)			all animals	no outliers
	compared to +10 time point, p values						paired vs. single	
	paired	single	all rats	paired	single	all rats		
10	x	x	x	x	x	x	+	+
20			0.045				+	+
30		-						+
40			0.044					+
50					-			+
60					-			+
70			0.014					+
80							+	+
90							+	+
100							+	+
110							+	+
120		0.030					+	+
130							+	+
140				-		-	+	+
150							+	+
160							+	+
170	-						+	+
180	-			-			+	+
190	-			-			+	+
200	-		-				+	+
210	-		-	-			+	+
220	-	-	-	-	-	-	+	+
230	-		-	-	-	-	+	+
240	-		-	-	-	-	+	+
250	-		-	-	-	-	+	+
260	-		-	-	-	-	+	+
270	-	-	-	-	-	-	+	+
280	-	-	-	-	-	0.050-	+	+
290	-	-	-	-	-	0.047-	+	+
300	0.026-	-	0.020-	0.035-	-	0.005-	+	+
310	0.013-	-	0.018-	0.013-	-	0.004-	0.015+	0.003+
320	0.008-	-	0.014-	0.016-	-	0.008-	0.012+	0.010+
330	0.022-	-	0.018-	0.050-	-	0.011-	+	+
340	0.009-	-	0.004-	0.046-	-	0.006-	+	+
350	0.035-	0.034-	0.002-	-	0.023-	0.004-	+	+
360	0.010-	-	0.003-	0.017-	0.048-	0.002-	+	0.037+
370	0.010-	0.031-	0.001-	0.017-	0.019-	0.001-	+	0.023+
380	0.003-	-	0.001-	0.005-	-	0.001-	+	0.016+
390	0.001-	0.040-	0.000-	0.005-	0.023-	0.000-	0.024+	0.017+
400	0.001-	-	0.000-	0.009-	0.025-	0.001-	0.005+	0.028+
410	0.001-	-	0.000-	0.005-	0.020-	0.000-	0.009+	0.011+
420	0.003-	0.031-	0.000-	0.009-	0.007-	0.000-	+	+
430	0.001-	-	0.000-	0.003-	0.019-	0.000-	0.048+	+
440	0.001-	0.040-	0.000-	0.011-	0.025-	0.001-	0.026+	+
450	0.002-	0.040-	0.000-	0.014-	0.009-	0.000-	0.021+	+
460	0.002-	0.036-	0.000-	0.014-	0.013-	0.001-	0.005+	0.025+
470	0.000-	0.012-	0.000-	0.006-	0.009-	0.000-	0.010+	0.010+
480	0.001-	0.002-	0.000-	0.009-	0.002-	0.000-	+	+
490	0.001-	0.002-	0.000-	0.012-	0.001-	0.000-	+	+
500	0.001-	0.002-	0.000-	0.006-	0.001-	0.000-	+	0.041+
510	0.001-	0.010-	0.000-	0.007-	0.003-	0.000-	0.025+	0.031+
520	0.001-	0.007-	0.000-	0.009-	0.001-	0.000-	0.023+	+
530	0.000-	0.002-	0.000-	0.002-	0.001-	0.000-	0.012+	0.048+
540	0.000-	0.000-	0.000-	0.001-	0.001-	0.000-	0.041+	+
550	0.000-	0.000-	0.000-	0.001-	0.001-	0.000-	+	0.033+
560	0.000-	0.000-	0.000-	0.001-	0.001-	0.000-	+	+
570	0.000-	0.000-	0.000-	0.001-	0.000-	0.000-	+	+
580	0.000-	0.000-	0.000-	0.000-	0.000-	0.000-	+	+
590	0.000-	0.000-	0.000-	0.002-	0.000-	0.000-	+	+
600	0.001-	0.000-	0.000-	0.002-	0.000-	0.000-		
	75%	61%	69%	73%	69%	68%	54/60 (90%)	59/60 (98%)

Table S5. Analysis of heart rate (HR) values during the first 10 min of silence at the beginning of playback session. Related to Figures 2 and S1; for paired (n = 30), single (n = 31) and all rats (n = 61), and these groups without outliers (i.e. with n = 21, 25, 46; respectively) evaluated for each 10-s-long interval. Left part; comparison of the results to the starting values for 10s time-interval; Wilcoxon's tests; (-) denotes each example of lower HR value than at 10 s time-interval. Right part; significant differences between paired and single groups – for all rats and after eliminating outliers; Mann-Whitney tests; (+) denotes higher HR value in single rats. Significant (< 0.05) p values are given.

time-interval [s]	all animals (61 rats)			no outliers (46 rats)			all animals	no outliers
	compared to +10 time point, p values							
	paired	single	all rats	paired	single	all rats	paired vs. single	
10	x	x	x	x	x	x	+	+
20	+	+	+					+
30	+	+	+					0.009+
40	+		+					+
50	+	0.037+	0.020+		+	+		0.009+
60	0.038+	+	0.005+	+	+	+		+
70	0.050+	+	0.028+	+	+	+	+	+
80	0.038+	+	0.013+		+	+		+
90	0.028+	0.013+	0.001+	+	0.041+	0.017+	+	+
100	0.011+	0.009+	0.000+	+	0.026+	0.007+	+	+
110	0.011+	0.022+	0.001+	+	+	0.020+	+	+
120	0.022+	0.028+	0.002+	+	+	0.041+		0.023+
130	0.028+	0.004+	0.000+	+	0.011+	0.006+	0.035+	0.001+
140	+	0.004+	0.001+	+	0.011+	0.005+	0.013+	0.001+
150	+	0.016+	0.002+	+	0.041+	0.032+	+	0.027+
160	+	0.005+	0.000+	+	0.013+	0.004+	0.027+	0.004+
170	0.046+	0.011+	0.001+	+	0.023+	0.017+	+	0.002+
180	+	0.003+	0.001+	+	0.007+	0.006+	0.011+	0.001+
190	+	0.003+	0.000+		0.006+	0.007+	0.005+	0.000+
200	+	0.012+	0.001+	+	0.033+	0.008+	+	0.040+
210	0.041+	0.041+	0.003+	+	+	0.032+	+	+
220	+	0.005+	0.001+	+	0.013+	0.005+	0.036+	0.015+
230	+	+	0.007+	+	+	0.049+	+	+
240	+	0.023+	0.006+		+	0.031+	0.011+	0.006+
250	+	0.016+	0.001+	+	0.034+	0.004+	+	+
260	+	+	0.015+	+		+	+	+
270	+	+	0.031+		+	+	+	0.022+
280	+	+	0.014+	+	+	+	+	+
290	+	+	0.031+		+	+	0.043+	0.003+
300	+	0.019+	0.008+	+	+	0.026+	0.012+	0.022+
310	+	0.045+	0.015+		+	+	0.039+	0.026+
320	+	+	+		+	+	+	+
330	+	+	0.039+		+	+	0.037+	0.025+
340	+	0.017+	0.006+	+	+	0.039+	0.016+	0.004+
350	+	+	0.039+	+		+		+
360	+	0.050+	0.020+		+	+	+	0.047+
370	+	+	+		+	+	+	0.043+
380	+	0.050+	0.008+	+	+	0.039+	+	+
390	+	0.045+	0.011+	+	+	+	+	+
400	+	0.020+	0.013+		+	+	0.009+	0.005+
410	+	+	+				0.026+	0.035+
420	+	+	+				+	+
430	+	+	0.013+	+	+	+	+	+
440	+	+	+	+	+	+	+	+
450	+	+	0.048+	+	+	+	+	+
460	+	+	0.041+		+	+	+	+
470	+	0.033+	0.027+		+	+	0.018+	0.014+
480	+	0.039+	0.018+	+	+	+	+	0.040+
490	+	+	+	+	+	+	+	+
500	+	0.038+	0.008+	+	+	0.023+	+	+
510	+	+	0.037+	+	+	+	+	+
520	+	0.050+	0.031+		+	+	0.032+	0.047+
530	+	+	0.017+		+	+	+	0.025+
540	+	0.036+	0.012+		+	+	0.043+	0.003+
550		0.024+	0.019+		+	0.047+	0.015+	0.013+
560	+	0.020+	0.008+	+	0.046+	0.042+	0.021+	0.012+
570	+	+	+		+	+	+	0.009+
580	+	+	+	+	+	+	+	+
590	+	+	+		+	+	+	0.032+
600		+	+				+	+
	97%	98%	100%	56%	86%	90%	52/60 (87%)	(100%)

Table S6. Analysis of numbers of USV during the first 10 min of silence at the beginning of playback session. Related to Figures 2 and S1; USV were emitted by paired (n = 30), single (n = 31) and all rats (n = 61), and these groups without outliers (n = 21, 25, 46; respectively) divided into 10-s-long intervals. Left part; comparison of the results to the starting values at 10 s time-interval; Wilcoxon; (+) denotes all instances of higher number of USV emitted than during 10 s time-interval. Right part; significant differences between paired and single groups – for all rats and after eliminating outliers; Mann-Whitney tests; (+) denotes higher value in single rats. Significant (< 0.05) p values are given.

time-interval [s] / group	**paired**	**single**	**all rats**	**paired**	**single**	**all rats**
**A. distance traveled**	**(n = 29)**	**(n = 31)**	**(n = 60)**	**(n = 29)**	**(n = 31)**	**(n = 60)**
		**50-kHz USV**			**50-kHz tone**	
-120 to 180	p < 0.05	p < 0.01	p < 0.001	p < 0.001	p < 0.01	p < 0.001
-60 to 60	p < 0.001	p < 0.01	p < 0.001	p < 0.001	p < 0.05	p < 0.001
-10 to 10	p < 0.01	p < 0.01	p < 0.001	p < 0.001	p = 0.28	p < 0.001
-120 to -100	p < 0.01	p = 0.54	p = 0.05	p = 0.52	p = 0.28	p = 0.89
-30 to -10	p = 0.87	p = 0.68	p = 0.94	p = 0.28	p = 0.09	p = 0.12
		**22-kHz USV**			**22-kHz tone**	
-120 to 180	p < 0.001	p < 0.001	p < 0.001	p = 0.15	p = 0.20	p = 0.63
-60 to 60	p < 0.01	p < 0.001	p < 0.001	p = 0.08	p = 0.30	p = 0.29
-10 to 10	p < 0.01	p < 0.001	p < 0.001	p = 0.08	p < 0.01	p = 0.42
-120 to -100	p = 0.58	p = 0.37	p = 0.95	p = 0.52	p < 0.05	p < 0.05
-30 to -10	p = 0.48	p = 0.25	p = 0.60	p = 0.38	p = 0.30	p = 0.95
**B. time in speaker's half**	**(n = 29)**	**(n = 31)**	**(n = 60)**	**(n = 29)**	**(n = 31)**	**(n = 60)**
		**50-kHz USV**			**50-kHz tone**	
-120 to 180	p < 0.001	p < 0.05	p < 0.001	p < 0.001	p < 0.001	p < 0.001
-60 to 60	p < 0.001	p = 0.43	p < 0.001	p < 0.01	p < 0.001	p < 0.001
-10 to 10	p < 0.05	p = 0.09	p < 0.01	p < 0.01	p < 0.05	p < 0.001
-120 to -100	p = 0.41	p = 0.18	p = 0.81	p = 0.17	p = 0.35	p = 0.88
-30 to -10	p = 0.30	p = 0.17	p = 0.82	p = 0.13	p = 0.88	p = 0.54
		**22-kHz USV**			**22-kHz tone**	
-120 to 180	p = 0.62	p < 0.05	p < 0.05	p < 0.05	p < 0.001	p < 0.001
-60 to 60	p = 0.96	p = 0.44	p = 0.49	p < 0.05	p < 0.001	p < 0.001
-10 to 10	p = 0.83	p < 0.05	p = 0.11	p = 0.81	p = 0.10	p = 0.29
-120 to -100	p = 0.28	p = 0.19	p = 0.67	p = 0.27	p = 0.77	p = 0.42
-30 to -10	p = 1.00	p = 0.90	p = 0.94	p = 0.93	p < 0.001	p < 0.01
**C. HR changes**	**(n = 30)**	**(n = 31)**	**(n = 61)**	**(n = 30)**	**(n = 31)**	**(n = 61)**
		**50-kHz USV**			**50-kHz tone**	
-120 to 180	p < 0.001	p < 0.001	p < 0.001	p < 0.001	p < 0.001	p < 0.001
-60 to 60	p < 0.001	p < 0.001	p < 0.001	p < 0.001	p < 0.01	p < 0.001
-10 to 10	p < 0.05	p < 0.001	p < 0.001	p < 0.05	p < 0.01	p < 0.001
-120 to -100	p = 0.27	p = 0.91	p = 0.64	p = 0.27	p = 0.66	p = 0.60
-30 to -10	p = 0.67	p = 0.67	p = 0.86	p = 0.53	p = 0.58	p = 0.67
		**22-kHz USV**			**22-kHz tone**	
-120 to 180	p < 0.001	p < 0.001	p < 0.001	p < 0.001	p < 0.001	p < 0.001
-60 to 60	p < 0.001	p < 0.001	p < 0.001	p < 0.001	p < 0.001	p < 0.001
-10 to 10	p < 0.05	p < 0.001	p < 0.001	p < 0.01	p < 0.05	p < 0.001
-120 to -100	p = 0.34	p = 0.88	p = 0.40	p = 0.27	p = 0.66	p = 0.43
-30 to -10	p = 0.20	p = 0.99	p = 0.45	p = 1.00	p = 0.54	p = 0.73
**D. number of USV**	**(n = 30)**	**(n = 31)**	**(n = 61)**	**(n = 30)**	**(n = 31)**	**(n = 61)**
		**50-kHz USV**			**50-kHz tone**	
-120 to 180	p < 0.001	p < 0.001	p < 0.001	p < 0.001	p < 0.001	p < 0.001
-60 to 60	p < 0.001	p < 0.001	p < 0.001	p < 0.001	p < 0.001	p < 0.001
-10 to 10	p < 0.001	p < 0.001	p < 0.001	p < 0.001	p < 0.001	p < 0.001
-120 to -100	p = 0.20	p = 0.73	p = 0.80	p = 0.20	p = 0.84	p = 0.30
-30 to -10	p = 0.70	p = 0.72	p = 0.64	p = 0.48	p = 0.52	p = 0.32
		**22-kHz USV**			**22-kHz tone**	
-120 to 180	p < 0.001	p < 0.001	p < 0.001	p < 0.01	p < 0.05	p < 0.001
-60 to 60	p < 0.05	p < 0.001	p < 0.001	p < 0.001	p = 0.18	p < 0.001
-10 to 10	p = 0.14	p < 0.05	p < 0.01	p < 0.05	p = 0.37	p < 0.05
-120 to -100	p = 0.90	p < 0.05	p = 0.14	p = 0.42	p = 0.25	p = 0.17
-30 to -10	p = 0.17	p = 0.35	p = 0.08	p = 0.21	p = 0.72	p = 0.83

Table S7. Evaluation of changes observed around playback. Related to Figures 3, 4, S2, S3, and S4. Analyzed were distance traveled (**A**), time spent in the speaker's half (**B**), heart rate (HR) levels (**C**), and the number of emitted USV (**D**) around the time of playback, i.e. 0 s time-interval (from -120 s to 180 s, -60 s to 60 s, -10 s to 10 s), as well as before playback (-120 s to -100 s, -30 s to -10 s) of ultrasounds (50-kHz USV, 50-kHz tones, 22-kHz USV, and 22-kHz tones). Significant (< 0.05) or exact p values following Friedman's tests of results obtained in paired, single, and all rats are given.

time-interval [s] / group	**paired**	**single**	**all rats**	**paired**	**single**	**all rats**
	**50-kHz USV and tones (together)**			**22-kHz USV and tones (together)**		
**A. distance traveled**	**(n = 58)**	**(n = 62)**	**(n = 120)**	**(n = 58)**	**(n = 62)**	**(n = 120)**
-120 to 180	p < 0.001	p < 0.001	p < 0.001	p < 0.01	p < 0.001	p < 0.001
-60 to 60	p < 0.001	p < 0.001	p < 0.001	p < 0.05	p < 0.001	p < 0.001
-10 to 10	p < 0.001	p < 0.01	p < 0.001	p = 0.32	p < 0.001	p < 0.001
-120 to -100	p = 0.07	p = 0.17	p = 0.29	p = 0.51	p = 0.12	p = 0.16
-30 to -10	p = 0.70	p = 0.23	p = 0.23	p = 0.72	p = 0.26	p = 0.77
**B. time in speaker's half**	**(n = 58)**	**(n = 62)**	**(n = 120)**	**(n = 58)**	**(n = 62)**	**(n = 120)**
-120 to 180	p < 0.001	p < 0.001	p < 0.001	p < 0.05	p < 0.001	p < 0.001
-60 to 60	p < 0.001	p < 0.001	p < 0.001	p = 0.16	p < 0.001	p < 0.001
-10 to 10	p < 0.001	p < 0.01	p < 0.001	p = 0.70	p < 0.01	p < 0.05
-120 to -100	p = 0.07	p = 0.08	p = 0.95	p = 0.67	p = 0.33	p = 0.51
-30 to -10	p < 0.05	p = 0.53	p = 0.84	p = 0.97	p < 0.01	p < 0.05
**C. HR changes**	**(n = 60)**	**(n = 62)**	**(n = 122)**	**(n = 60)**	**(n = 62)**	**(n = 122)**
-120 to 180	p < 0.001	p < 0.001	p < 0.001	p < 0.001	p < 0.001	p < 0.001
-60 to 60	p < 0.001	p < 0.001	p < 0.001	p < 0.001	p < 0.001	p < 0.001
-10 to 10	p < 0.01	p < 0.001	p < 0.001	p < 0.001	p < 0.001	p < 0.001
-120 to -100	p = 0.16	p = 0.89	p = 0.54	p < 0.05	p = 0.81	p = 0.12
-30 to -10	p = 0.73	p = 0.92	p = 0.86	p = 0.35	p = 0.73	p = 0.86
**D. number of USV**	**(n = 60)**	**(n = 62)**	**(n = 122)**	**(n = 60)**	**(n = 62)**	**(n = 122)**
-120 to 180	p < 0.001	p < 0.001	p < 0.001	p < 0.001	p < 0.001	p < 0.001
-60 to 60	p < 0.001	p < 0.001	p < 0.001	p < 0.001	p < 0.001	p < 0.001
-10 to 10	p < 0.01	p < 0.001	p < 0.001	p < 0.001	p < 0.05	p < 0.001
-120 to -100	p = 0.09	p = 0.86	p = 0.40	p = 0.10	p = 0.06	p = 0.19
-30 to -10	p = 0.81	p = 0.96	p = 0.92	p = 0.45	p = 0.71	p = 0.94

Table S8. Evaluation of changes observed around playback with USV and tones pooled and analyzed together. Related to Figure 4. Examined were distance traveled (**A**), time spent in the speaker's half (**B**), heart rate (HR) levels (**C**), and the number of emitted USV (**D**) around the time of playback, i.e. 0 s time-interval (-120 s to 180 s, -60 s to 60 s, -10 s to 10 s), as well as before playback (-120 s to -100 s, -30 s to -10 s) of ultrasounds – 50-kHz USV and tones (analyzed together), 22-kHz USV and tones (also analyzed together). Significant (< 0.05) or exact p values following Friedman's tests of results obtained in paired, single, and all rats are given.

	vs. 0 s time-interval			vs. +10 s time-interval			paired/ single	vs. 0 s time-interval			vs. +10 s time-interval			paired/ single
time	paired	single	all rats	paired	single	all rats		paired	single	all rats	paired	single	all rats	
[s]	50-kHz USV							50-kHz tone						
-120	0.008-	0.002-	0.000-				+	0.001-	-	0.001-	+	+	+	+
-110	-	0.001-	0.000-	+				0.004-	0.023-	0.000-	+	+	+	+
-100	0.018-	0.005-	0.000-	+		+	+	0.000-	-	0.000-		+	+	0.006+
-90	0.023-	0.000-	0.000-	+				0.001-	-	0.002-	+	0.011+	+	0.019+
-80	0.005-	0.001-	0.000-	+				-	-	0.005-	+	+	0.037+	
-70	0.033-	0.000-	0.000-	+				0.002-	-	0.000-	+	+	+	+
-60	0.011-	0.006-	0.000-	+	+	+	+	0.003-	-	0.001-	+	0.033+	0.029+	+
-50	0.047-	0.002-	0.000-	+		+		0.001-	0.006-	0.000-	+		+	
-40	0.035-	0.001-	0.000-	+		+	+	0.002-	0.025-	0.000-	+	+	+	+
-30	0.012-	0.000-	0.000-	+			+	0.000-	-	0.001-		+	+	0.009+
-20	0.016-	0.005-	0.000-	+		+	+	0.000-	0.000-	0.000-				+
-10	0.002-	0.004-	0.000-				+	0.000-	0.038-	0.000-		+	+	+
0	x	x	x	0.005+	0.003+	0.000+	+	x	x	x	0.000+	0.007+	0.000+	
10	0.005-	0.003-	0.000-	x	x	x	+	0.000-	0.007-	0.000-	x	x	x	+
20	0.014-	0.000-	0.000-	+			+	0.001-	0.001-	0.000-	+		+	
30	-	0.019-	0.017-	0.031+	+	+		0.000-	0.009-	0.000-	+	+	+	+
40	-	0.014-	0.002-	+	+	+	+	0.000-	0.010-	0.000-	+	+	+	
50	-	0.007-	0.005-	0.017+	+	+		0.000-	0.017-	0.000-	+	+	+	
60	-	0.024-	0.004-	+	+	0.040+	+	0.004-	0.003-	0.000-	+	+	+	
70	0.007-	0.010-	0.000-	+	+	+	+	0.003-	0.028-	0.000-	+	+	+	+
80	-	0.002-	0.001-	+		+	+	0.000-	0.005-	0.000-	+		+	
90	0.011-	0.005-	0.000-	+			+	0.001-	0.011-	0.000-	+	+	+	
100	0.005-	0.000-	0.000-				+	0.001-	0.001-	0.000-	+		+	
110	0.018-	0.002-	0.000-	+		+		0.000-	0.004-	0.000-				+
120	0.024-	0.002-	0.000-	+		+	+	0.000-	0.000-	0.000-				+
130	0.043-	0.034-	0.003-	+	+	+	+	0.000-	0.004-	0.000-				+
140	0.035-	0.002-	0.000-	+		+	+	0.000-	0.002-	0.000-				+
150	0.010-	0.000-	0.000-	+				0.000-	0.014-	0.000-		+		0.030+
160	0.028-	0.004-	0.000-	+			+	0.000-	0.001-	0.000-				+
170	0.020-	0.006-	0.000-	+	+	+	+	0.000-	0.002-	0.000-				+
180	0.041-	0.003-	0.000-	+		+	+	0.000-	0.000-	0.000-				
	22-kHz USV							22-kHz tone						
-120				0.000+	0.001+	0.000+			-	-	+	+	+	
-110				0.003+	0.000+	0.000+			-	-	+	+	+	+
-100				0.001+	0.001+	0.000+				0.033	+	0.011+	0.007+	+
-90		-		0.000+	0.001+	0.000+	0.039		-		+	+	+	+
-80				0.003+	0.002+	0.000+			-		+	+	+	
-70		-	-	0.005+	0.008+	0.000+					+	+	0.016+	+
-60				0.004+	0.000+	0.000+		-				+	+	+
-50	-			0.023+	0.000+	0.000+	+	-				+	+	+
-40	-			0.025+	0.000+	0.000+	+				+	+	+	+
-30	-			0.016+	0.000+	0.000+	+	-		-		+	+	+
-20	-	-	-	0.004+	0.002+	0.000+					+	0.044+	0.041+	+
-10				0.001+	0.000+	0.000+		-				0.010+	+	0.006+
0	x	x	x	0.007+	0.000+	0.000+		x	x	x		0.025+	+	+
10	0.007-	0.000-	0.000-	x	x	x			0.025-	-	x	x	x	
20	-	-	0.006-	+	0.019+	0.016+		-	-	-		0.034+	+	+
30	-	0.015-	0.033-	0.037+	0.010+	0.001+			-		+	+	0.036+	+
40	-		-	+	0.000+	0.000+	+		-		+	0.023+	0.026+	+
50		-		0.000+	0.000+	0.000+			-		+	+	0.023+	
60		-		0.037+	0.001+	0.000+			-	-		+	+	+
70	-			+	0.000+	0.000+	+				+	0.028+	0.021+	+
80	-		-	0.002+	0.000+	0.000+	+		-	-	+	+	+	
90				0.037+	0.000+	0.000+			-		+	+	0.020+	
100		-	-	+	0.000+	0.000+					+	+	+	+
110	-	-	-	0.030+	0.000+	0.000+			-		+	+	+	
120	-	-	-	+	0.000+	0.000+	+		-		+	+	+	+
130				+	0.000+	0.000+			-		+	+	+	+
140	-			+	0.000+	0.000+	+		-		+	+	+	+
150	-		-	+	0.000+	0.000+	+		-		+	+	+	
160	-	-	-	+	0.001+	0.003+	+		-		+	+	0.049+	+
170	-		-	0.045+	0.000+	0.000+	+	0.007	-		0.037+		+	
180	-		-	+	0.000+	0.000+	+	0.041	-		+	+	0.044+	

Table S9. Analysis of distance traveled during playback session. Related to Figures 3, 4, S2-4; for paired (n = 29), single (n = 31), and all rats (n = 60), before (from -120 s to -10 s), during (at 0 s), and after (10 s to 180 s) presentation of 50-kHz USV, 50-kHz tones, 22-kHz USV and 22-kHz tones; significant p values are given after comparing the results with those at 0 and 10 s time-intervals with Wilcoxon tests; for "0 s", (-) denotes a value lover than at 0 s; for "+10 s", (+) denotes a value higher than at 10 s. Also, under "paired/single", the differences in distance traveled between these groups are evaluated with Mann-Whitney comparisons; significant p values are given; (+) shows a higher value in single rats.

	vs. 50% value			vs. +10 s time-interval			paired/	vs. 50% value			vs. +10 s time-interval			paired/
**time**	paired	single	all rats	paired	single	all rats	single	paired	single	all rats	paired	single	all rats	single
[s]	**50-kHz USV**							**50-kHz tone**						
-120				0.001-	0.020-	0.000-	+		+		0.001-	0.047-	0.000-	0.045+
-110				0.001-	0.008-	0.000-	+		+	+	0.005-	-	0.001-	+
-100				0.002-	0.017-	0.000-		+	+	+	0.022-	0.015-	0.001-	+
-90				0.005-	-	0.002-		+		+	0.011-	0.002-	0.000-	
-80		+		0.004-	-	0.002-	+	+	+	+	-	0.007-	0.002-	
-70		+	+	0.006-	-	0.008-	+	+	+	+	0.048-	0.012-	0.001-	
-60		+	+	0.028-	-	0.013-	+	+			0.028-	0.002-	0.000-	
-50		+	+	0.022-	-	0.007-	+				0.016-	0.005-	0.000-	+
-40		+		0.006-	-	0.010-	+		+		0.006-	0.012-	0.000-	+
-30		+		0.006-	-	0.013-	+		+	+	0.004-	0.019-	0.000-	+
-20		+	+	0.012-	-	0.016-	+		+	+	0.017-	0.025-	0.001-	+
-10	+	+	+	0.030-	-	0.011-	+	+	+	+	0.027-	0.033-	0.002-	+
0	+	+	+	0.047-	-	0.008-		+	0.000+	0.001+	0.007-	-	0.002-	+
10	0.004+	+	0.001+	x	x	x		0.001+	0.000+	0.000+	x	x	x	+
20	0.003+	0.040+	0.000+		-			0.001+	0.000+	0.000+				
30	0.006+	0.037+	0.001+	-	-	-		0.035+	0.000+	0.000+	-	-	-	+
40	0.024+	0.022+	0.001+	-	-	-		+	0.016+	0.010+	-	-	0.016-	+
50	0.030+	0.024+	0.002+	-	-	-	+	+	+	+	-	0.041-	0.005-	+
60	+	0.008+	0.049+	0.008-		0.049-	+	+	+	+	-	0.007-	0.003-	
70	+	0.032+	+	0.003-		0.031-	+	+	+	+	-	0.007-	0.003-	
80	+	+	+	0.019-	-	0.044-	+	+		+	-	0.002-	0.001-	
90	+	+	+	-	-	-	+	+			0.042-	0.001-	0.000-	
100		+	+	0.019-	-	-	+				0.016-	0.002-	0.000-	+
110		+		0.012-	-	0.022-	+				0.005-	0.004-	0.000-	+
120	+	+	+	0.028-	-	0.030-	+				0.003-	0.005-	0.000-	+
130		+	+	0.028-	-	0.026-	+				0.001-	0.002-	0.000-	+
140	+	+	+	0.035-	-	0.009-	+		+		0.000-	0.023-	0.000-	+
150	+	+	+	-	-	0.013-			+		0.003-	0.016-	0.000-	+
160	+	+	+	-	-	0.037-			+		0.003-	0.016-	0.000-	+
170	+	+	+	0.038-	-	0.012-					0.005-	0.006-	0.000-	+
180	+	+	+	0.011-	-	0.006-					0.002-	0.004-	0.000-	+
	**22-kHz USV**							**22-kHz tone**						
-120		+		-	-	0.009-	+		+		-	0.008-	0.007-	+
-110		+		-	-	0.014-	+		+		-	0.023-	0.028-	+
-100		+		-	-	0.034-	0.039+		+		-	0.018-	0.028-	+
-90		+		-	-	0.021-	+		+		-	-	-	+
-80		+		-	-	0.020-	+		+		-	-	0.046-	+
-70		+		-	-	-	+				-	0.015-	0.016-	+
-60		+	+	-	-	-	+				-	0.004-	0.024-	
-50		+	+	-	-	-	+				-	0.001-	0.008-	
-40	+	+	+	-	-	-	+	+				0.001-	0.014-	
-30	+	+	+	-	-	-	+	+			-	0.002-	0.015-	
-20	+	+	+	-	-	-	+		+	+	-	0.033-	-	+
-10	+	+	+	-	-	-	+	+	0.041+	+	-	-	-	+
0	+	+	+	-	-	-	+		0.002+	0.029+	-	-	-	0.022+
10	+	0.019+	0.036+	x	x	x	+	+	0.005+	0.028+	x	x	x	0.040+
20	+	0.030+	0.045+	-	-	-	+	+	+	0.048+	0.028	0.028-	-	+
30	+	+	+	-	-	-	+	0.031+	+	0.011+	0.008	0.022-	-	
40	+	0.016+	+	-	-	-	+	0.018+	+	0.008+	0.023	-		
50	+	0.048+	+	-	-	-	+	+	+	0.038+		0.017-	-	
60	+	+	+	-	-	-	+	+	+	+		-	-	+
70	+	+	+	-	-	-	+	+	+	+		0.034-	-	+
80	+	+	+	-	-	-	+	+	+	+	-	0.007-	-	+
90		+	+	-	-	-	+				-	0.016-	-	+
100		+	+	-	-	-	+				-	0.005-	-	+
110	+		+	-	0.021-	0.023-			+	+	-	0.044-	-	+
120		+	+	-	-	-	+		+	+	-	-	-	+
130	+	+	+	-	-	-	+		+	+	-	-	-	+
140				-	0.014-	-			+	+	-	-	-	+
150				-	0.010-	0.019-			+	+	-	-	-	+
160	+			-	0.013-	-			+	+	-	-	-	+
170	+	+	+	-	0.026-	-			+	+	-	-	-	+
180		+	+	-	-	-	+		+		-	0.019-	0.011-	+

Table S10. Analysis of time spent in the speaker's half of the cage during playback session. Related to Figures 3, 4, S2-4; for paired (n = 29), single (n = 31), and all rats (n = 60), before (from -120 s to -10 s), during (at 0 s), and after (10 s to 180 s) presentation of 50-kHz USV, 50-kHz tones, 22-kHz USV and 22-kHz tones; significant p values are given after comparing the results with 50% value of chance level and values at 10 s time-intervals with Wilcoxon tests; for "50%", (+) denotes residing longer in the speaker's half; for "+10 s", (-) denotes a value lower than at 10 s. Also, under "paired/single", the differences in time spent in the speaker's half between these groups are evaluated with Mann-Whitney comparisons; significant p values are given; (+) shows a higher value in single rats.

	vs. -10 s time-interval			vs. 0 s time-interval			paired/ single	vs. -10 s time-interval			vs. 0 s time-interval			paired/ single
time	paired	single	all rats	paired	single	all rats		paired	single	all rats	paired	single	all rats	
[s]	**50-kHz USV**							**50-kHz tone**						
-120		+	+	+	0.033+	0.020+	+	+	+	0.024+	0.041+	0.008+	0.001+	+
-110		+	+	+	0.009+	0.002+	+	+	+	+	0.024+	+	0.005+	+
-100		+	+	+	0.017+	0.003+	+	+	+	0.013+	0.032+	0.006+	0.000+	+
-90		+	+	+	0.003+	0.001+	+	+	+	+	+	0.024+	0.003+	+
-80		+		+	0.045+	0.026+	+	+	+	+	+	+	+	0.041+
-70		+		+	+	+	+	+		+	+	+	+	+
-60		+	+	+	+	+	+	+	+	+	+	+	0.007+	+
-50		+		+	+	+	+	0.043+	+	+	0.001+	+	0.001+	+
-40				+	+	+	+	+	+	+	+	+	0.023+	+
-30				+	+	+		+		+	+	+	+	+
-20	+		+	+	+	+		+	+	+	+	+	+	+
-10	x	x	x	0.033+	+	0.015+		x	x	x	+	+	+	+
0	0.033		0.015	x	x	x	+				x	x	x	+
10		0.006+	+	+	0.000+	0.000+	0.045+	+	+	0.045+	0.011+	0.001+	0.000+	+
20	+	0.011+	0.007+	0.000+	0.000+	0.000+	+	0.024+	+	0.013+	0.002+	0.004+	0.000+	+
30	0.033+	0.004+	0.000+	0.000+	0.000+	0.000+	+	0.011+	+	0.002+	0.000+	0.000+	0.000+	+
40	+	0.003+	0.002+	0.001+	0.000+	0.000+	+	0.033+	+	0.012+	0.001+	0.006+	0.000+	+
50	0.045+	0.004+	0.001+	0.000+	0.000+	0.000+	+	+	+	0.037+	0.003+	0.008+	0.000+	+
60	0.039+	0.027+	0.002+	0.000+	0.009+	0.000+		+	0.034+	0.024+	+	0.002+	0.000+	0.029+
70	+	0.015+	0.003+	0.000+	0.004+	0.000+		+	0.033+	+	+	0.001+	0.001+	0.014+
80	+	0.005+	0.003+	0.000+	0.002+	0.000+	+	+	+	0.028+	+	0.004+	0.001+	+
90	+	+	0.030+	0.001+	0.044+	0.000+		+	+	+	0.003+	0.016+	0.000+	+
100	+	+	+	0.007+	+	0.014+		+	+	+	+	+	+	+
110	+	+	+	0.016+	0.031+	0.002+	+	+			+		+	+
120	+	+	+	0.010+	+	0.002+					+	+	+	+
130	+	+	+	0.024+	+	0.004+	+				+			+
140		+		+	+	+		+			+			+
150	+		+	+	+	+		+			+		+	+
160		+		+	+	+	+							+
170		+		+	+	+	+				+			+
180		+			+	+	+							+
	**22-kHz USV**							**22-kHz tone**						
-120	+	0.004+	0.006+	0.000+	0.000+	0.000+	+	+	+	+	0.006+	0.002+	0.000+	+
-110	+	0.025+	+	0.000+	0.000+	0.000+	+		+	+	0.006+	0.001+	0.000+	+
-100	+	0.003+	0.013+	0.000+	0.000+	0.000+	+	+	+	+	0.006+	0.007+	0.000+	+
-90	+	0.046+	0.013+	0.000+	0.000+	0.000+	+	+	+	+	0.001+	0.007+	0.000+	+
-80	+	0.016+	0.015+	0.000+	0.000+	0.000+	+	+	+	+	0.001+	0.002+	0.000+	+
-70	+	+	+	0.000+	0.000+	0.000+	+	+	+	+	0.002+	0.000+	0.000+	+
-60	+	+	+	0.001+	0.000+	0.000+	+	+			0.005+	0.020+	0.000+	+
-50	+	+	+	0.000+	0.000+	0.000+	+	+			0.003+	0.027+	0.000+	+
-40	+	+	+	0.000+	0.000+	0.000+	+	+			0.005+	0.014+	0.000+	+
-30	+	+	+	0.000+	0.000+	0.000+	+	+			0.001+	0.009+	0.000+	+
-20	+	+	+	0.001+	0.000+	0.000+	+	+	+	+	0.002+	0.003+	0.000+	+
-10	x	x	x	0.001+	0.000+	0.000+	+	x	x	x	0.000+	0.003+	0.000+	+
0	0.001	0.000	0.000	x	x	x	+	0.000	0.000	0.000	x	x	x	+
10	0.004	0.003	0.000	+	0.046+	+	+	0.001	0.003	0.000				+
20	0.043		0.023	+	0.016+	0.010+	+		0.015	0.007	+	+	+	+
30				+	0.001+	0.000+	+				0.030+	+	0.006+	+
40		+		0.009+	0.000+	0.000+	+				0.021+	+	0.005+	+
50	+	+	+	0.002+	0.000+	0.000+	+				+	+	0.025+	+
60	+	+	+	0.001+	0.000+	0.000+	+			0.045	+	+	0.017+	+
70	+	+	+	0.000+	0.001+	0.000+					+	+	0.024+	+
80	+	+	+	0.000+	0.002+	0.000+					+	+	+	+
90		+		0.006+	0.000+	0.000+	+	+			0.032+	+	0.023+	
100				0.004+	0.001+	0.000+	+				+	+	0.043+	+
110		+		0.008+	0.001+	0.000+	+		0.011	0.016	+	+	+	
120	0.039			0.033+	0.001+	0.000+	+			0.033	0.043+	+	+	+
130		+		+	0.002+	0.000+	+				0.041+	+	+	
140		+	+	0.045+	0.001+	0.000+	+			0.030	+	+	+	+
150		+		+	0.001+	0.000+	+	0.045		0.009	+	+	+	+
160		+		+	0.002+	0.001+	+			0.009	+	+	+	+
170		+		+	0.000+	0.000+	+		0.044		0.014+	+	0.036+	+
180		+		+	0.001+	0.001+	+				+	+	+	+

Table S11. Analysis of heart rate (HR) values during playback session. Related to Figures 3, 4, S2-4; for paired (n = 30), single (n = 31), and all rats (n = 61), analyzed before (from -120 s to -10 s), during (at 0 s), and after (10 s to 180 s) presentation of 50-kHz USV, 50-kHz tones, 22-kHz USV and 22-kHz tones; significant p values are given after comparing the results with those at -10-s- and 0 s time-intervals with Wilcoxon tests; (+) denotes a higher HR value then at -10 s and 0 s in respective columns. Also, under "paired/single", the differences in HR values between these groups are evaluated with Mann-Whitney comparisons; significant p values are given; (+) shows a higher value for single rats.

	vs. -10 s time-interval			vs. 0 s time-interval			paired/ single	vs. -10 s time-interval			vs. 0 s time-interval			paired/ single
**rats:**	paired	single	all rats	paired	single	all rats		paired	single	all rats	paired	single	all rats	
**time**	**50-kHz USV**							**50-kHz tone**						
-120	+	+	+	0.000-	0.000-	0.000-	+		+	+	0.000-	0.000-	0.000-	+
-110	+		+	0.000-	0.000-	0.000-	+	+	+	+	0.000-	0.000-	0.000-	+
-100				0.000-	0.000-	0.000-	+	+		+	0.000-	0.000-	0.000-	+
-90	+			0.000-	0.000-	0.000-	+	+	+	+	0.000-	0.000-	0.000-	+
-80	+	+	+	0.000-	0.000-	0.000-	+	+			0.000-	0.000-	0.000-	+
-70	+	+	+	0.000-	0.000-	0.000-	+	+			0.000-	0.000-	0.000-	+
-60	+	+	+	0.000-	0.000-	0.000-	+				0.000-	0.000-	0.000-	+
-50	+		+	0.000-	0.000-	0.000-	+	+	+	+	0.000-	0.000-	0.000-	+
-40	+			0.000-	0.000-	0.000-	+	+			0.000-	0.000-	0.000-	+
-30	+		+	0.000-	0.000-	0.000-	+				0.000-	0.000-	0.000-	+
-20	+	+	+	0.000-	0.000-	0.000-	+				0.000-	0.000-	0.000-	+
-10	x	x	x	0.000-	0.000-	0.000-	+	x	x	x	0.000-	0.000-	0.000-	+
0	0.000+	0.000+	0.000+	x	x	x		0.000+	0.000+	0.000+	x	x	x	
10	0.000+	0.000+	0.000+			0.023	+	0.000+	0.000+	0.000+	-		-	+
20	0.000+	0.000+	0.000+	-			+	0.000+	0.000+	0.000+	0.050-	-	0.020-	+
30	0.000+	0.000+	0.000+	-		-	+	0.000+	0.000+	0.000+	0.022-	0.011-	0.001-	+
40	0.000+	0.000+	0.000+	0.034-	-	-	+	0.000+	0.002+	0.000+	0.001-	0.003-	0.000-	+
50	0.000+	0.000+	0.000+	0.013-	-	0.004-	+	0.001+	0.004+	0.000+	0.003-	0.000-	0.000-	+
60	0.001+	0.000+	0.000+	0.000-	-	0.000-	+	0.000+	0.002+	0.000+	0.001-	0.001-	0.000-	
70	0.000+	0.000+	0.000+	0.000-	0.026-	0.000-	+	0.011+	0.005+	0.000+	0.000-	0.000-	0.000-	+
80	0.002+	0.001+	0.000+	0.000-	0.002-	0.000-	+	0.002+	+	0.003+	0.000-	0.000-	0.000-	+
90	0.006+	0.001+	0.000+	0.000-	0.001-	0.000-	+	0.001+	0.048+	0.000+	0.000-	0.000-	0.000-	+
100	0.014+	0.012+	0.000+	0.000-	0.001-	0.000-	+	+	+	0.009+	0.000-	0.000-	0.000-	+
110	0.034+	0.007+	0.001+	0.000-	0.000-	0.000-	+	+	+	0.037+	0.000-	0.000-	0.000-	+
120	0.026+	0.016+	0.001+	0.000-	0.001-	0.000-	+	+	+	+	0.000-	0.001-	0.000-	+
130	0.019+	0.011+	0.001+	0.000-	0.000-	0.000-	+	+	+	+	0.000-	0.000-	0.000-	+
140	+	+	0.048+	0.000-	0.000-	0.000-	+	+	+	+	0.000-	0.000-	0.000-	
150	+	+	0.020+	0.000-	0.000-	0.000-	+		+	+	0.000-	0.000-	0.000-	+
160	+	+	+	0.000-	0.000-	0.000-	+	+	+	+	0.000-	0.000-	0.000-	+
170	0.046+	+	0.008+	0.000-	0.000-	0.000-	+	+	+	+	0.000-	0.000-	0.000-	+
180	+	+	0.037+	0.000-	0.000-	0.000-	+	+		+	0.000-	0.000-	0.000-	
	**22-kHz USV**							**22-kHz tone**						
-120	+			-	0.038-	0.030-		+	+	+	-	-	-	+
-110	+	+	+	-		-	+	+	+	+	-	-	-	+
-100	+		+	-	-	-	+	+	+	+	-		-	+
-90	+		+	-	-	-					-	0.003-	0.001-	+
-80	+			-	0.028-	0.005-		+			-	0.005-	0.002-	+
-70	+			-	-	0.023-	+	+		+	-	-	-	+
-60	+			0.044-	-	0.016-	+				0.008-	-	0.003-	+
-50	+		+	-	-	-		+			-	0.013-	0.002-	+
-40	+			-	-	0.040-		+			-	0.039-	0.008-	+
-30	+			-	-	0.035-	+	+			-	-	0.021-	+
-20				0.050-	-	0.008-	+				0.016-	-	0.009-	+
-10	x	x	x	-	-	0.018-	+	x	x	x	0.050-	-	0.021-	0.046+
0	+	+	0.018+	x	x	x		0.050+	+	0.021+	x	x	x	+
10	+		+	-	0.011-	0.029-		+	+	+	-		-	+
20	+	+	0.033+	-				+	+	+		-	-	
30	+	0.018+	0.005+	-			+	0.013+	+	0.002+				
40	+	0.017+	0.004+	-			+	+	+	+	-	-	-	+
50	0.011+	0.004+	0.000+		0.030		+	+		+	-	-	-	+
60	+	+	0.027+	-				+		+	-	-	-	+
70	0.004+	0.026+	0.000+					+	+	+	-	-	-	+
80	0.015+	+	0.013+					+			-	0.024-	0.021-	
90	0.028+	+	0.025+	-	-	-		+		+	-	-	-	+
100	0.015+		+	-	-	-		+	+	+	-	-	-	+
110	0.011+		+	-	0.018-	-		+	+	+	-	-	-	+
120	+	+	+	-	-	-	+	+			-	0.019-	0.003-	
130	0.008+	+	0.024+	-			+	+			-	0.035-	0.015-	
140	+	+	+	-	-	-	+				-	0.033-	0.002-	+
150	+	+	+	-	-	-	+	+			-	0.021-	0.007-	+
160	+	+	+	-		-	+				-	0.024-	0.001-	+
170	+	+	+	-		-	+	+			-	0.005-	0.002-	
180	0.039+		+	-	-	-	+				-	0.010-	0.001-	+

Table S12. Analysis of the number of USV during playback session. Related to Figures 3, 4, S2-4; for paired (n = 30), single (n = 31), and all rats (n = 61), before (from -120 s to -10 s), during (at 0 s), and after (10 s to 180 s) presentation of 50-kHz USV, 50-kHz tones, 22-kHz USV and 22-kHz tones; significant p values are given after comparing the results with those at -10-s- and 0 s time-intervals, Wilcoxon tests; for "-10 s", (+) denotes a value higher than at -10 s; for "0 s", (-) denotes a value lower than at 0 s. Under "paired/single", the differences between these groups are evaluated, Mann-Whitney; significant p values are given; (+) denotes higher value for single rats.

rats: n =	paired [58]	single [62]	all rats [120]	paired [58]	single [62]	all rats [120]	paired / single	paired [58]	single [62]	all rats [120]	paired [58]	single [62]	all rats [120]	paired / single
**A. distance traveled**														
**50-kHz, USV and tones**								**22-kHz, USV and tones**						
**time**	**vs. 0 s time-interval**			**vs. +10 s time-interval**				**vs. 0 s time-interval**			**vs. +10 s time-interval**			
-120	0.000-	0.002-	0.000-	+	+	+	+	0.044	-		0.001+	0.007+	0.000+	
-110	0.001-	0.000-	0.000-	0.050+		+					0.005+	0.001+	0.000+	+
-100	0.000-	0.003-	0.000-	+	+	+	+			0.022	0.000+	0.000+	0.000+	+
-90	0.000-	0.001-	0.000-	+	+	+	+		-		0.001+	0.003+	0.000+	
-80	0.000-	0.000-	0.000-	+	+	+					0.006+	0.002+	0.000+	
-70	0.000-	0.000-	0.000-	+		+					0.003+	0.002+	0.000+	+
-60	0.000-	0.002-	0.000-	+	+	+	+				+	0.000+	0.000+	+
-50	0.000-	0.000-	0.000-	+		+		-			+	0.000+	0.000+	+
-40	0.000-	0.000-	0.000-	+	+	+	+				0.020+	0.000+	0.000+	+
-30	0.000-	0.001-	0.000-		+	+	0.020+	-			0.038+	0.000+	0.000+	+
-20	0.000-	0.000-	0.000-				+				0.017+	0.000+	0.000+	+
-10	0.000-	0.001-	0.000-		+		+				+	0.000+	0.000+	+
0	x	x	x	0.000+	0.000+	0.000+		x	x	x	+	0.000+	0.000+	+
10	0.000-	0.000-	0.000-	x	x	x	+	-	0.000-	0.000-	x	x	x	
20	0.000-	0.000-	0.000-	+		+	+	-	-	0.030-	+	0.002+	0.006+	+
30	0.001-	0.000-	0.000-	0.025+	+	0.015+	+		0.034-	-	0.024+	0.003+	0.000+	
40	0.000-	0.000-	0.000-	0.024+	+	0.023+	+		-		+	0.000+	0.000+	+
50	0.001-	0.000-	0.000-	0.008+	+	0.019+			-		0.000+	0.000+	0.000+	
60	0.001-	0.000-	0.000-	0.010+	+	0.006+	+		-	-	+	0.000+	0.000+	
70	0.000-	0.001-	0.000-	+	+	+	+				0.037+	0.000+	0.000+	+
80	0.000-	0.000-	0.000-	+		+			-	-	0.008+	0.000+	0.000+	+
90	0.000-	0.000-	0.000-	+		+	+		-		0.008+	0.000+	0.000+	
100	0.000-	0.000-	0.000-	+			+				+	0.000+	0.000+	+
110	0.000-	0.000-	0.000-	+			+		-	-	0.037+	0.000+	0.000+	
120	0.000-	0.000-	0.000-	+			+	-	-	-	+	0.000+	0.000+	+
130	0.000-	0.000-	0.000-	+	+	+	+				+	0.000+	0.000+	+
140	0.000-	0.000-	0.000-	+			+				+	0.000+	0.000+	+
150	0.000-	0.000-	0.000-				+	-			+	0.001+	0.002+	+
160	0.000-	0.000-	0.000-				+	-	-	-	+	0.001+	0.000+	+
170	0.000-	0.000-	0.000-	+			+		-		0.005+	0.002+	0.000+	
180	0.000-	0.000-	0.000-	+			+		-		0.028+	0.000+	0.000+	
**B. time in the speaker's half**														
	**vs. 50% value**			**vs. +10 s time-interval**				**vs. 50% value**			**vs. +10 s time-interval**			
-120	0.046	+		0.000-	0.002-	0.000-	+	0.032	+		0.033-	0.001-	0.000-	+
-110				0.000-	0.001-	0.000-	+	0.041	+		-	0.007-	0.001-	0.038+
-100				0.000-	0.000-	0.000-			+		-	0.011-	0.002-	0.035+
-90	+			0.000-	0.001-	0.000-			+		-	0.014-	0.005-	+
-80	+	+	+	0.001-	0.004-	0.000-			+		-	0.009-	0.002-	+
-70	+	+	+	0.001-	0.010-	0.000-	+		+		-	0.006-	0.005-	+
-60	+		+	0.002-	0.001-	0.000-			+	+	-	0.004-	0.005-	+
-50		+		0.001-	0.002-	0.000-	+		+		-	0.002-	0.002-	+
-40		+		0.000-	0.020-	0.000-	+	+	+	+	-	0.001-	0.013-	
-30		+	+	0.000-	0.047-	0.000-	0.013+	+	+	+	-	0.001-	0.011-	+
-20		+	+	0.001-	0.037-	0.000-	+	+	+	+	-	0.011-	0.047-	+
-10	+	+	+	0.002-	0.013-	0.000-	+	+	0.030+	+	-	-	-	+
0	0.029+	0.002+	0.000+	0.002-	0.013-	0.000-	+	+	0.000+	0.008+	-	-	-	0.023+
10	0.000+	0.000+	0.000+	x	x	x		+	0.000+	0.003+	x	x	x	0.009+
20	0.000+	0.000+	0.000+					+	0.006+	0.005+		-	-	+
30	0.001+	0.000+	0.000+	-	-	-	+	+	0.015+	0.003+		0.008-	-	+
40	0.007+	0.002+	0.000+	0.042-	-	0.022-	+	+	0.007+	0.003+		-	-	+
50	+	0.004+	0.001+	0.019-	-	0.007-	+	+	0.022+	0.012+		0.039-	-	+
60	+	0.010+	0.013+	0.003-	-	0.000-	+	+	0.021+	0.020+		-	-	+
70	+	0.020+	0.040+	0.002-	-	0.000-	+	+	+	+	-	0.016-	-	+
80	+	+	+	0.005-	0.004-	0.000-		+	+	+	-	0.003-	0.026-	+
90	+	+	+	0.005-	0.008-	0.000-			+		-	0.006-	0.012-	+
100		+	+	0.001-	0.011-	0.000-	+		+		-	0.001-	0.012-	+
110		+		0.000-	0.008-	0.000-	+		+	+	-	0.003-	0.019-	+
120		+		0.000-	0.008-	0.000-	+		+	+	-	0.016-	0.019-	+
130		+		0.000-	0.004-	0.000-	+		+	+	-	0.028-	0.027-	+
140		+		0.000-	0.006-	0.000-	+		+		-	0.014-	0.012-	+
150	+	+	+	0.000-	0.004-	0.000-	+		+		-	0.002-	0.003-	+
160	+	+	+	0.001-	0.008-	0.000-	+		+	+	-	0.003-	0.027-	+
170	+	+	+	0.001-	0.002-	0.000-	+		+	+	-	0.004-	0.024-	+
180				0.000-	0.002-	0.000-	+		+	+	-	0.004-	0.004-	+

Table S13. Analysis of distance traveled (A) and time spent (B). Related to Figures 4 and Table S8; for paired, single, and all rats [numbers of data-points in brackets], before (-120 s to -10 s), during (0 s), and after (10 s to 180 s) presentation of 50-kHz USV and tones as well as 22-kHz USV and tones combined respectively. For distance, comparisons with 0 s and 10 s time-intervals; (-) denotes a value lover than at 0 s; (+) denotes a value higher than at 10 s. For time, (+) denotes residing longer in the speaker's half; (-) denotes a value lower than at 10 s, Wilcoxon; "paired/single" shows differences between these groups, Mann-Whitney; (+) a higher value for single rats. Significant p values are given.

rats: n =	paired [60]	single [62]	all rats [122]	paired [60]	single [62]	all rats [122]	paired / single	paired [60]	single [62]	all rats [122]	paired [60]	single [62]	all rats [122]	paired / single
**C. HR changes**														
	**50-kHz, USV and tones**							**22-kHz, USV and tones**						
**time**	**vs. -10 s time-interval**			**vs. 0 s time-interval**				**vs. -10 s time-interval**			**vs. 0 s time-interval**			
-120	+	0.035+	0.037+	0.028+	0.001+	0.000+	0.038+	+	0.003+	0.003+	0.000+	0.000+	0.000+	+
-110	+	0.039+	0.017+	0.005+	0.001+	0.000+	+		0.012+	+	0.000+	0.000+	0.000+	0.026+
-100	+	0.023+	0.007+	0.004+	0.000+	0.000+	+	+	0.005+	0.028+	0.000+	0.000+	0.000+	+
-90	+	0.008+	0.009+	0.026+	0.000+	0.000+	0.048+	+	+	0.046+	0.000+	0.000+	0.000+	+
-80		+	+	+	0.004+	0.004+	0.031+	+	0.022+	0.017+	0.000+	0.000+	0.000+	+
-70		+		0.021+	+	0.009+	+	+	0.034+	0.020+	0.000+	0.000+	0.000+	+
-60		+	+	0.015+	0.021+	0.001+	+	+	+	+	0.000+	0.000+	0.000+	+
-50	+	+	+	0.003+	+	0.002+	+	+	+	+	0.000+	0.000+	0.000+	+
-40				+	+	0.042+	+	+	+	+	0.000+	0.000+	0.000+	+
-30	+		+	+	+	0.041+	+	+	+	+	0.000+	0.000+	0.000+	+
-20	+		+	0.018+	+	0.011+	+	+	+	+	0.000+	0.000+	0.000+	+
-10	x	x	x	+	+	0.006+	+	x	x	x	0.000+	0.000+	0.000+	+
0			0.006	x	x	x	+	0.000	0.000	0.000	x	x	x	+
10	+	0.005+	0.010+	0.001+	0.000+	0.000+	0.010+	0.000	0.000	0.000		+	+	+
20	0.013+	0.006+	0.000+	0.000+	0.000+	0.000+	+	0.014	0.008	0.000	0.046+	+	0.006+	+
30	0.001+	0.001+	0.000+	0.000+	0.000+	0.000+	+	0.036			0.004+	0.001+	0.000+	+
40	0.012+	0.002+	0.000+	0.000+	0.000+	0.000+	+				0.000+	0.000+	0.000+	+
50	0.015+	0.003+	0.000+	0.000+	0.000+	0.000+	+		+		0.000+	0.000+	0.000+	+
60	0.025+	0.002+	0.000+	0.000+	0.000+	0.000+	+				0.000+	0.000+	0.000+	+
70	+	0.001+	0.001+	0.000+	0.000+	0.000+	+				0.000+	0.001+	0.000+	+
80	+	0.001+	0.000+	0.000+	0.000+	0.000+	+				0.000+	0.001+	0.000+	+
90	0.039+	+	0.010+	0.000+	0.001+	0.000+	+				0.001+	0.002+	0.000+	+
100	+	+	+	0.008+	+	0.002+	+				0.001+	0.002+	0.000+	+
110	+	+	+	0.019+	+	0.012+	+			0.013	0.002+	0.024+	0.000+	+
120	+	+	+	0.026+	+	0.012+	+	0.031		0.006	0.002+	0.004+	0.000+	+
130	+			+	+	+	+				0.005+	0.011+	0.000+	+
140				+		+	+				0.008+	0.005+	0.000+	+
150	+			+		+	+	0.009		0.009	0.017+	0.015+	0.001+	+
160				+	+	+	+	0.020		0.012	0.029+	0.026+	0.002+	+
170				+	+	+	+	0.038			0.002+	0.002+	0.000+	+
180		0.034			+		+	0.022		0.025	0.023+	0.006+	0.000+	+
**D. number of USV**														
	**vs. -10 s time-interval**			**vs. 0 s time-interval**				**vs. -10 s time-interval**			**vs. 0 s time-interval**			
-120	+	+	+	0.000-	0.000-	0.000-	+	+		+	-	0.020-	0.006-	+
-110	+	+	+	0.000-	0.000-	0.000-	+	+	+	+	-	-	-	+
-100	+		+	0.000-	0.000-	0.000-	+	0.023+	+	+	-	-	0.039-	+
-90	+	+	+	0.000-	0.000-	0.000-	+	+			0.039-	0.004-	0.000-	+
-80	+		+	0.000-	0.000-	0.000-	+	+			0.019-	0.000-	0.000-	+
-70	+			0.000-	0.000-	0.000-	+	+		+	0.035-	-	0.005-	+
-60		+	+	0.000-	0.000-	0.000-	+				0.001-	0.032-	0.000-	+
-50	+	+	+	0.000-	0.000-	0.000-	+	+			0.016-	0.014-	0.000-	0.044+
-40	+			0.000-	0.000-	0.000-	+	+			0.021-	0.026-	0.001-	+
-30				0.000-	0.000-	0.000-	+	+			0.025-	0.022-	0.001-	+
-20		+		0.000-	0.000-	0.000-	0.039+				0.002-	0.041-	0.000-	0.024+
-10	x	x	x	0.000-	0.000-	0.000-	+	x	x	x	0.006-	-	0.000-	0.016+
0	0.000+	0.000+	0.000+	x	x	x	+	0.006+	+	0.000+	x	x	x	
10	0.000+	0.000+	0.000+	-			+	0.032+	+	+	-	0.043-	0.015-	+
20	0.000+	0.000+	0.000+	0.037-	-	0.040-	+	0.006+	+	0.012+	-			+
30	0.000+	0.000+	0.000+	0.024-	-	0.004-	+	0.004+	0.004+	0.000+	-			+
40	0.000+	0.000+	0.000+	0.000-	0.019-	0.000-	+	0.022+	0.024+	0.001+	-			+
50	0.000+	0.000+	0.000+	0.000-	0.000-	0.000-	+	0.005+	0.010+	0.000+	-			0.041+
60	0.000+	0.000+	0.000+	0.000-	0.000-	0.000-	+	0.035+	+	0.028+	-	-	-	
70	0.000+	0.000+	0.000+	0.000-	0.000-	0.000-	+	0.008+	+	0.001+				+
80	0.000+	0.000+	0.000+	0.000-	0.000-	0.000-	+	0.007+	+	0.043+	-	-	-	
90	0.000+	0.000+	0.000+	0.000-	0.000-	0.000-	+	0.010+	+	+	-	-	-	+
100	0.003+	0.002+	0.000+	0.000-	0.000-	0.000-	+	+		+	-	-	0.016-	+
110	0.009+	0.005+	0.000+	0.000-	0.000-	0.000-	+	0.013+		+	-	0.039-	-	
120	0.041+	0.021+	0.002+	0.000-	0.000-	0.000-	+	+			0.019-	0.046-	0.002-	+
130	0.006+	0.011+	0.000+	0.000-	0.000-	0.000-	+	0.014+	+	+	-	-	0.026-	+
140	0.042+	+	0.027+	0.000-	0.000-	0.000-	+	+			0.008-	0.014-	0.000-	+
150	+	+	0.047+	0.000-	0.000-	0.000-	+	+		+	-	0.013-	0.002-	+
160	+	+	+	0.000-	0.000-	0.000-	+				0.008-	0.016-	0.000-	+
170	+	+	0.034+	0.000-	0.000-	0.000-	+	+		+	0.040-	-	0.005-	+
180	0.016+	+	0.045+	0.000-	0.000-	0.000-	+	+			0.027-	0.008-	0.000-	+

Table S14. Analysis of HR levels (C) and numbers of USV emitted (D). Related to Figures 4 and Table S8; for paired, single, and all rats [numbers of data-points in brackets], before (-120 s to -10 s), during (0 s), and after (10 s to 180 s) presentation of 50-kHz USV and tones as well as 22-kHz USV and tones combined. Significant p values are given for comparisons with -10 s and 0 s time-intervals. For HR, (+) denotes a value lover than at -10 s or 0 s. For USV, (+) denotes a value higher than at -10 s, (-) a value lower than at 0 s, Wilcoxon; “paired/single” shows differences between these groups, Mann-Whitney; significant p values are given; (+) denotes a higher value for single rats.

time	50-kHz USV vs. 22-kHz USV		50-kHz tones vs. 22-kHz tones		50-kHz USV and tones vs. 22-kHz USV and tones		50-kHz USV vs. 22-kHz USV	50-kHz tones vs. 22-kHz tones	50-kHz USV and tones vs. 22-kHz USV and tones
groups	paired	single	paired	single	paired	single	all rats		all rats
**A. distance traveled**									
-120				0.044+		+		0.017+	+
-110	+		0.017+	+	+	+		0.005+	+
-100		+		+		+	+	+	+
-90		+		0.018+		0.026+		0.024+	+
-80			+	+	+	+		+	+
-70	+	+	+	+	+	+	+	+	+
-60		+	0.019+	+	+	+		0.041+	+
-50	+		+	+	+		+	+	+
-40	+		+	+	+	+	+	+	+
-30	+			+	+	+		+	+
-20		+					+		
-10		+		+		+		+	+
0	0.011+	0.000+	0.000+	0.002+	0.000+	0.000+	0.000+	0.000+	0.000+
10	0.005+	0.000+	+	+	0.005+	0.000+	0.000+	0.032+	0.000+
20	0.011+	0.007+	0.005+	+	0.000+	0.016+	0.000+	0.013+	0.000+
30	0.020+	0.004+	0.015+	+	0.001+	0.005+	0.000+	0.029+	0.000+
40	+	+	+	+	+	+	0.017+	+	0.014+
50	+	+		+	+	+	+	+	0.017+
60	+	0.004+	0.003+	+	0.006+	0.004+	0.007+	0.003+	0.000+
70	+	+	+	+	+	+	+	+	+
80	+	+	+	+	+	+	+	+	+
90		+	+	+	+	+	+	+	+
100		+	+		+			+	+
110	+	+		+	+	+	0.047+		+
120	+	+	+		+	+	0.041+		+
130	+	+		+	+	+	+	+	+
140	+	+	+	+	+	+	+	+	+
150	+		+	+	+	+	+		+
160	+	+	+		+	+	+		+
170	+	+	+	+		+	+		+
180	+	+	+	+		+	+		+
**B. time in the speaker's half**									
-120	+			+		+		+	+
-110	+		+	+	+			+	
-100	+	0.031	+	+	+			+	+
-90	+		+		+		+	+	+
-80	+		+	+	+	+	+	+	+
-70	+	+	+	+	+	+	+	+	+
-60	+		+		+		+	+	+
-50	+		+	+	+			+	+
-40				+		+			
-30				+		+		+	
-20				+		+			
-10	+								
0	+		+		+		+	+	+
10	+		0.017+	+	0.006+	+	+	0.025+	0.040+
20	+		+	+	0.010+	+	+	0.022+	0.011+
30	+	+	+	0.034+	+	+	+	+	0.046+
40	+			+	+	+	+		+
50	+			+	+	+	+		+
60		+	+				+		
70		+	+		+	+	+		+
80	+	+	+		+		+		+
90	+	+	+		+	+	+	+	+
100		+	+		+	+	+		+
110		+							
120	+	+					+		
130		+					+		
140	+	+			+		+		
150	+	+			+	+	+		+
160	+	+			+	+	+		+
170	+	+			+		+		
180	+				+				

Table S15. Analysis of comparisons between the effects on distance traveled (A) and the time spent in the speaker's half (B) of 50-kHz vs. 22-kHz playback of USV, tones, as well as USV and tones combined. Related to Figures 3, 4, and S2. Playbacks were presented during 0 s time-intervals to paired (n = 29), single (n = 31) and all rats (n = 60); significant p values are given, all Wilcoxon tests; (+) denotes a higher value for rats presented with 50-kHz playback.

time	50-kHz USV vs. 22-kHz USV		50-kHz tones vs. 22-kHz tones		50-kHz USV and tones vs. 22-kHz USV and tones		50-kHz USV vs. 22-kHz USV	50-kHz tones vs. 22-kHz tones	50-kHz USV and tones vs. 22-kHz USV and tones
**groups**	**paired**	**single**	**paired**	**single**	**paired**	**single**	**all rats**		**all rats**
**C. HR levels**									
-120			+	+	+	+		+	+
-110	+	+	+	+	+	+	+	+	+
-100	+		+	+	+	+	+	+	+
-90	+	+	+	+	+	+	+	+	+
-80				+		+			
-70	+								
-60		+	+	+		+	+	+	+
-50			+	+	+	+		+	+
-40	+		+	+	+	+		+	+
-30	+	+		+	+	+	+	+	+
-20	+	+		+	+	+	+	+	+
-10	+	+		+	+	+	+	+	+
0	0.008+	0.005+	+	0.033+	0.003+	0.001+	0.000+	0.008+	0.000+
10	0.000+	0.000+	0.006+	0.001+	0.000+	0.000+	0.000+	0.000+	0.000+
20	0.001+	0.001+	+	0.003+	0.000+	0.000+	0.000+	0.001+	0.000+
30	0.001+	0.002+	+	0.016+	0.000+	0.000+	0.000+	0.002+	0.000+
40	0.009+	0.024+	+	0.040+	0.004+	0.002+	0.001+	0.017+	0.000+
50	0.015+	0.042+	+	+	0.005+	0.005+	0.002+	0.014+	0.000+
60	0.016+	+	+	0.042+	0.008+	0.010+	0.008+	0.023+	0.000+
70	0.021+	0.034+	+	0.023+	0.009+	0.002+	0.002+	0.010+	0.000+
80	0.030+	0.020+	+	0.046+	0.008+	0.002+	0.001+	0.009+	0.000+
90	0.028+	+	+	+	0.028+	0.008+	0.004+	+	0.001+
100	+	+	+	0.019+	+	0.011+	0.033+	0.044+	0.003+
110	0.018+	+	+	+	+	0.028+	0.005+	+	0.006+
120	0.004+	+	+	+	0.033+	0.027+	0.002+	+	0.002+
130	0.032+	+		+	+	+	0.031+	+	0.031+
140	+		+	+	+	+	+	+	+
150	0.010+	+	+	+	0.013+	+	0.012+	+	0.006+
160	0.035+	+		+	+	+	0.046+	+	0.034+
170	+	+		+	+	+	+	+	+
180	+	+		+	+	+	+		+
**D. number of USV**									
-120		+		+		+			
-110			+		+			+	
-100									0.044
-90			+	+		+		+	
-80		0.013+		+		0.048+	+	+	+
-70		+					+		
-60		+	+		+		+		+
-50			+	+	+	+		+	+
-40			+						
-30				+					
-20	+	+		0.044	+		+	0.035	
-10			+						
0	0.000+	0.000+	0.000+	0.000+	0.000+	0.000+	0.000+	0.000+	0.000+
10	0.000+	0.000+	0.000+	0.000+	0.000+	0.000+	0.000+	0.000+	0.000+
20	0.000+	0.000+	0.000+	0.000+	0.000+	0.000+	0.000+	0.000+	0.000+
30	0.000+	0.001+	0.002+	0.001+	0.000+	0.000+	0.000+	0.000+	0.000+
40	0.001+	0.005+	0.004+	0.004+	0.000+	0.000+	0.000+	0.000+	0.000+
50	0.014+	0.022+	0.005+	0.007+	0.000+	0.001+	0.001+	0.000+	0.000+
60	0.050+	0.005+	0.001+	0.020+	0.000+	0.000+	0.001+	0.000+	0.000+
70	+	+	0.043+	0.013+	0.030+	0.002+	0.025+	0.001+	0.000+
80	+	+	+	+	0.017+	0.035+	0.041+	0.009+	0.001+
90	+	0.042+	+	+	+	0.027+	0.012+	+	0.003+
100	+	0.020+	0.038+	+	0.029+	0.024+	0.012+	+	0.002+
110		0.016+	+	+	+	0.040+	+	+	0.038+
120	+	0.033+	+	+	0.048+	0.017+	0.013+	+	0.003+
130		+	+	+	+	+	+	+	+
140	+	+	0.018+	+	+	+	+	+	0.024+
150	+	+		+		+	+	+	+
160			+	+	+	+		+	+
170	+		+	+	+	+	+	+	+
180	+		0.018+	+	0.018+	+	+	+	0.011+

Table S16. Analysis of comparisons between the effects on heart rate, HR (C) and the number of emitted USV (D) of 50-kHz vs. 22-kHz playback of USV, tones, as well as USV and tones combined. Related to Figures 3, 4, and S2. Playbacks were presented during 0 s time-intervals to paired (n = 30), single (n = 31) and all rats (n = 61); significant p values are given, all Wilcoxon tests; (+) denotes a higher value for rats presented with 50-kHz playback.

signals	USV vs. tone		USV vs. tone		USV vs. tone			USV vs. tone		USV vs. tone		USV vs. tone	
	50-kHz	50-kHz	22-kHz	22-kHz	50-kHz	22-kHz		50-kHz	50-kHz	22-kHz	22-kHz	50-kHz	22-kHz
groups	paired	single	paired	single	all rats			paired	single	paired	single	all rats	
**A. distance traveled**								**B. time in the speaker's half**					
time	n = 29	n = 31	n = 29	n = 31	n = 60	n = 60		n = 29	n = 31	n = 29	n = 31	n = 60	n = 60
-120			+	+		+		+			+		
-110			+	+		0.041+							
-100	+		+	0.033					0.041		+		
-90	+	0.000	+		0.010	+					+		
-80			+	+	0.023	+					+		
-70			+		0.032				+		+		+
-60	0.048		0.027+			+			+		+	+	+
-50			+	+		+			+	+	0.015+	+	+
-40				+					+		0.041+	+	+
-30	+	0.007	+	+		+				+	+		+
-20	+	+	+		+				+	+	+		+
-10			0.010+			+							
0		+	+			+		+		+			
10		+		0.013		0.004				+			
20											+		+
30	+	+			+			+			+		
40		+			+			+	+	0.036	+	+	
50	+		+		+	+		+	+		+	+	
60		+	+		+	+			+		+	+	+
70		+	+	+		+			+	+		+	+
80		+		+	+	+		+	+		+	+	+
90				+	0.041			+	+		+	+	+
100			+					+	+	+	+	+	+
110	+				+			+	+	+		+	
120	+	+			+			+	+	+		+	
130	+	+	+	+	+	+		+	+	+		+	+
140	+	+		+	+	+		+		+		+	
150	0.041+		0.033		+			+	+	+		+	
160	+	+			+			+	+	+	0.019	+	
170	+	+		+	+			+	+	+		+	
180	+	+		+	+			+	+	+	+	+	+
**C. HR levels**								**D. number of USV**					
	n = 30	n = 31	n = 30	n = 31	n = 61	n = 61		n = 30	n = 31	n = 30	n = 31	n = 61	n = 61
-120								+		+			
-110			+							+			+
-100			+	+		+							
-90			+			+				+	+		+
-80	+								+				
-70	+												
-60			+	+		+			+	+		+	
-50			+	+		+				+	+		+
-40	+									+			
-30	+							+					
-20	+							+	+			+	
-10	+				+								
0	+			0.010		0.009				+			
10	+	+			+			+	+	+	0.025	0.037+	
20	+	+			+			+	+	+	+	+	+
30	+				+			+	0.025+		+	0.012+	
40	+	+		+	+			+	0.050+	+	+	0.016+	+
50	0.045+		+	+	+	+		+	0.006+	+	0.037+	0.010+	0.006+
60	0.020+		+	+	+	+		+	+	+	+	+	+
70	0.015+		+		+	+		0.013+	0.037+	+	+	0.001+	0.021+
80	+		+		+	+		+	0.002+	+	+	0.001+	+
90	+				+			+	+	+	+	+	+
100	+				+			+	+	+		+	
110	0.032+	+		+	+			+	+	+		+	+
120	+	+		+	+			+	+		+	+	+
130	0.035+	+		+	+			+	+	+	+	+	+
140	+	+		+	+	+		+	+	+	+	+	+
150	+			+	+	+		+	+	+	+	+	+
160	+			+	+	+			+	+	+	+	+
170	+	+		+	+			+	+	+	0.042+	+	0.035+
180	+	+			+				+	+	+	+	+

Table S17. Statistical evaluation of comparisons between the effects of natural ultrasonic vocalizations (USV) vs. artificially-generated tones. Related to Figures 3, 4, S3, and S4; for both 50- and 22-kHz types, on the distance traveled (**A**), time spent in the speaker's half of the cage (**B**), heart rate (HR, **C**), and number of USV emitted in response (**D**). Both USV- and tones-playback recordings were presented during 0 s time-intervals to paired, single, and all rats (the numbers of animals per group are given); significant p values are shown, all Wilcoxon tests; (+) denotes a higher value for rats presented with natural USV-playback.
